# Bidirectional Mamba-2 boosts EEG super-resolution via regression and diffusion

**DOI:** 10.1093/bioinformatics/btag169

**Published:** 2026-04-15

**Authors:** Ugo Lomoio, Pietro Lió, Pietro Hiram Guzzi, Pierangelo Veltri

**Affiliations:** Department of Surgical and Medical Sciences, Magna Graecia University, Catanzaro 88100, Italy; DIMES, University of Calabria, Rende 87036, Italy; Computer Science and Technology, University of Cambridge, Cambridge CB2 1TN, United Kingdom; Department of Surgical and Medical Sciences, Magna Graecia University, Catanzaro 88100, Italy; DIMES, University of Calabria, Rende 87036, Italy

## Abstract

**Motivations:**

Electroencephalography (EEG) is a non-invasive method that records brain electrical activity from scalp electrodes, offering millisecond temporal resolution but limited spatial detail due to sparse sensor layouts.

**Results:**

We present DiBiMa-EEGSR, a bidirectional Mamba-2 diffusion framework for spatio-temporal EEG super-resolution that reconstructs high-resolution signals from standard low-density recordings without additional hardware. The method formulates super-resolution as conditional generative inference and integrates a diffusion process with a bidirectional state-space backbone to model long-range temporal dependencies with linear complexity. Conditioning on low-resolution inputs, electrode positions and task labels enables anatomically coherent and context-aware reconstruction. A one-step sampling strategy substantially reduces inference time while preserving fidelity. Across two public benchmarks, the approach improves reconstruction accuracy, spatial coherence and spectral preservation over convolutional, transformer-based and prior diffusion models in both spatial and temporal upsampling tasks, providing a scalable pathway toward high-resolution electrophysiological imaging.

**Availability and implementation:**

Code to reproduce ablation experiments, training and evaluation of the proposed BiMa and DiBiMa EEGSR models are available at https://github.com/UgoLomoio/DiBiMa-EEGSR.git. Model weights are available at https://huggingface.co/Ugo96/DiBiMa-EEGSR while an interactive demo for EEG spatial super-resolution using our models can be found at https://huggingface.co/spaces/Ugo96/DiBiMa-EEGSR-Demo.

## 1 Introduction

Electroencephalography (EEG) is a non-invasive neuroimaging technique with millisecond temporal resolution, and remains central to clinical neurophysiology, cognitive neuroscience and brain–computer interface (BCI) research (Burle et al. 2015, Zhang and Chen 2025). This temporal fidelity, however, comes with an intrinsic limitation: scalp recordings provide only coarse spatial information, constraining the localization of neural generators and the fine-grained characterization of cortical activity (Burle et al. 2015, Kwon et al. 2019, Wang et al. 2026).

**Figure 1:**
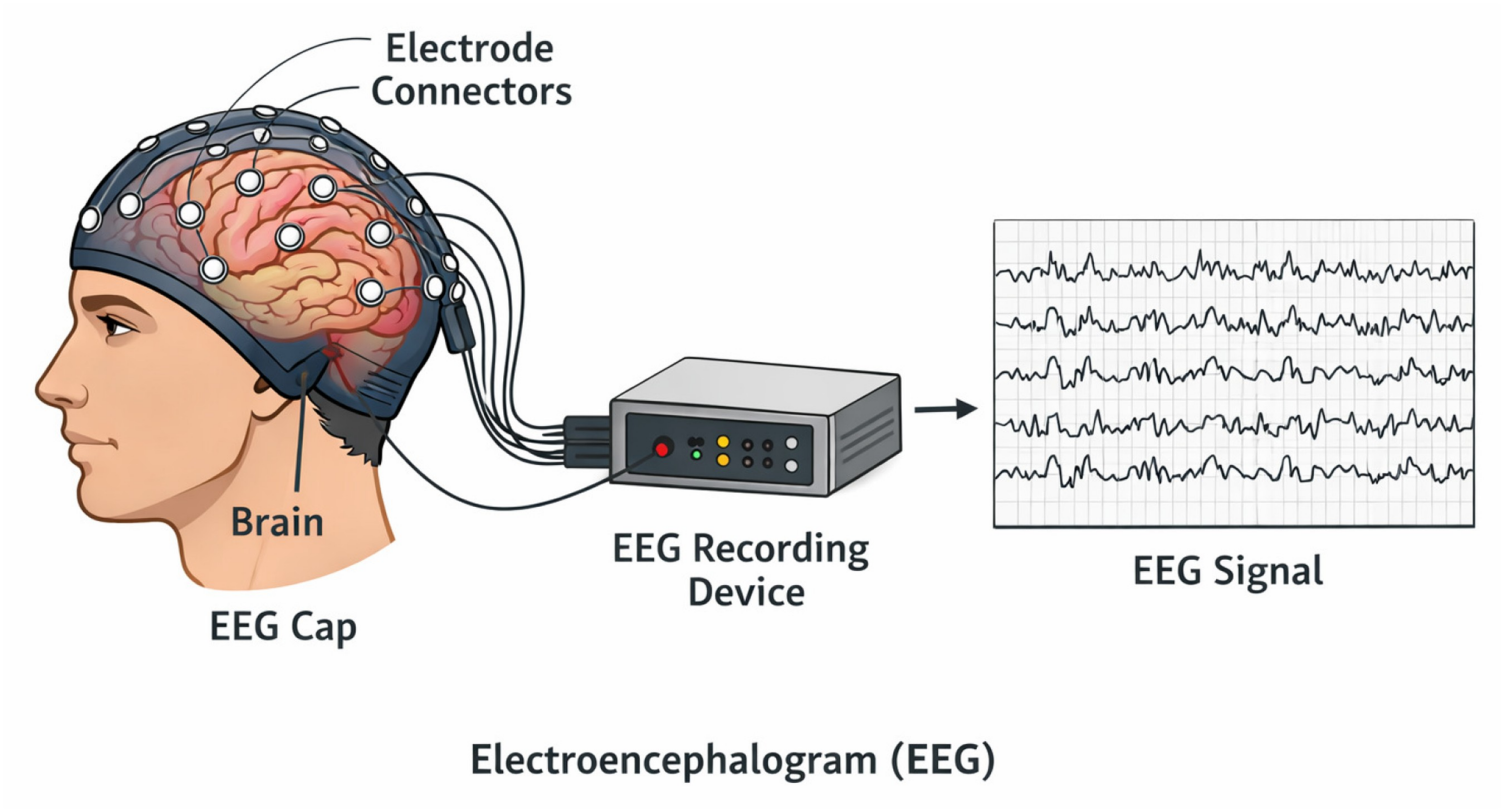
Schematic overview of electroencephalography (EEG) signal acquisition. Scalp electrodes arranged in an EEG cap record the aggregate electrical activity generated by cortical neuronal populations. Signals are conveyed to a recording system for amplification and digitization, producing multichannel EEG waveforms that reflect ongoing brain activity.

Standard EEG montages typically use 32–64 electrodes, yielding effective spatial resolution on the order of centimetres—often insufficient when fine spatial discrimination is required. Figure 1 summarizes the basic principles of EEG acquisition.

High-density (HD) EEG systems (e.g. 256 channels) can partially mitigate this limitation, but their cost, setup burden and reduced practicality continue to hinder routine use in clinical workflows and large-scale studies (El-Fiqi et al. 2016).

To enhance spatial detail without additional hardware, spatio-temporal EEG super-resolution (SR) has emerged as a computational strategy for reconstructing high-resolution (HR) signals from low-resolution (LR) recordings using data-driven models (Tang et al. 2021). The task is inherently ill-posed: multiple HR configurations can map to the same LR observation. Accurate reconstruction therefore demands models that capture dependencies across electrode space and signal time. EEG exhibits structured spatio-temporal correlations shaped by volume conduction, electrode geometry and distributed cortical sources whose dynamics unfold across extended temporal horizons (Kundu et al. 2024). These properties motivate architectures that jointly encode spatial topology and long-range temporal evolution.

Early EEG SR approaches, including interpolation schemes and convolutional neural networks (CNNs), largely relied on local spatial or temporal receptive fields, limiting their ability to represent long-range structure and non-stationary neural dynamics (Corley and Huang 2018, Tang et al. 2021). Transformer-based models improve global dependency modelling, but their quadratic attention cost and memory footprint restrict scalability to long sequences and dense channel settings (Pfeffer et al. 2024). Diffusion-based generative models have further advanced EEG SR by casting reconstruction as probabilistic denoising, enabling uncertainty-aware synthesis and often improving physiological plausibility. Yet existing diffusion pipelines typically adopt generic U-Net or attention-heavy denoisers that do not fully exploit the sequential structure and inductive properties of electrophysiological signals.

**Figure 2:**
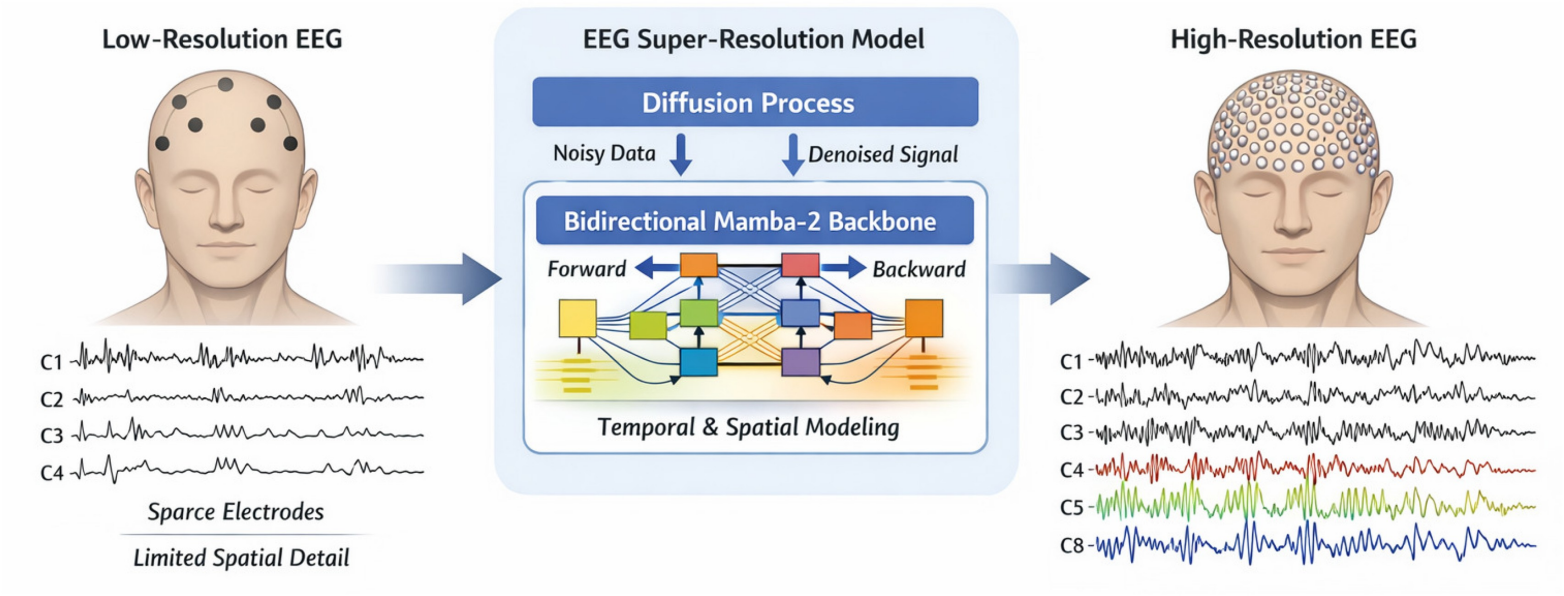
Conceptual overview of the proposed Bidirectional Mamba-2 Diffusion framework for spatio-temporal EEG super-resolution. Conventional low-resolution (LR) EEG acquired from sparse electrode configurations provides limited spatial detail, constraining interpretation and downstream analysis. Our framework formulates super-resolution as conditional generative inference, combining a diffusion-based reconstruction process with a bidirectional Mamba-2 state-space backbone. By jointly modelling long-range temporal dynamics and inter-channel relationships, the model reconstructs high-resolution (HR) EEG that preserves clinically relevant structure, including waveform morphology, oscillatory rhythms and spatial patterns, without additional acquisition hardware.

Here, we propose a Bidirectional Mamba-2 Diffusion model for spatio-temporal EEG super-resolution, designed to address these limitations with an efficient, sequence-aware generative framework (Fig. 2).

Our approach integrates diffusion-based reconstruction with a bidirectional Mamba-2 backbone—a structured state-space model that captures long-range temporal dependencies with linear computational complexity. By incorporating both forward and backward temporal context during denoising, each time point can be inferred from its full temporal neighborhood, a natural fit for non-causal electrophysiological signals. This design enables joint modelling of temporal dynamics and inter-channel structure while remaining computationally efficient for long EEG sequences.

Relative to CNN-based SR methods, the proposed framework moves beyond local receptive fields by representing global temporal structure through state-space dynamics. Compared with Transformer architectures, the Mamba-2 backbone avoids the quadratic scaling of self-attention, enabling efficient training and inference on high-resolution signals. In contrast to existing diffusion-based EEG SR models, our structured state-space denoiser introduces an inductive bias better aligned with sequential electrophysiology, improving reconstruction stability, sample efficiency and robustness across subjects. Consequently, the Bidirectional Mamba-2 Diffusion model produces physiologically plausible HR EEG reconstructions while maintaining resilience to noise and inter-subject variability.

By unifying probabilistic generative modelling with an efficient bidirectional state-space architecture, this work strengthens the methodological basis of EEG super-resolution. The resulting framework provides a scalable, hardware—independent route to enhanced spatial resolution in EEG analysis, with implications for clinical neurophysiology, cognitive neuroscience and next-generation BCI systems. More broadly, it reframes spatio-temporal EEG SR as structured generative inference, enabling high-resolution electrophysiological imaging from standard low-density recordings.

The main contributions of this work are 3-fold. First, we introduce a bidirectional Mamba-2 diffusion framework for spatio-temporal EEG super-resolution that couples probabilistic generative modelling with a structured state-space architecture. By embedding the diffusion process within a Mamba-2 backbone and conditioning on task labels, electrode-position embeddings and low-resolution inputs, the model captures long-range temporal dependencies and inter-channel structure, enabling context-aware and subject-adaptive reconstruction.

Second, the bidirectional Mamba-2 denoiser supports non-causal, sequence-level inference with linear complexity, providing a scalable alternative to attention-based and convolutional designs. Building on consistency diffusion modelling (Daras et al. 2023) and rectified diffusion sampling (Zhou et al. 2024), we adopt a one-step inference strategy that initializes reverse diffusion from a noise-perturbed, upsampled signal, substantially reducing sampling cost while improving alignment between training and inference.

Third, we frame EEG super-resolution as structured conditional inference. By modelling the conditional distribution of HR EEG given low-density observations and contextual information, the framework yields physiologically plausible, subject-specific reconstructions from standard recordings without additional hardware.

This paper is organized as follows. Related work subection discusses main related work. The Problem Formulation section presents the problem formulation, the diffusion-based reconstruction process and the integration of the bidirectional Mamba-2 state-space model within the denoising architecture. We then detail training and inference procedures and implementation choices, followed by experimental evaluation and comparative analyses against existing super-resolution approaches.

### 1.1 Related work

Early efforts in EEG super-resolution (SR) were grounded in classical signal-processing approaches, most notably Spherical Splines Interpolation (SSI) (Freeden 1984). SSI models scalp potentials on a geodesic surface using spherical basis functions, enabling interpolation of missing electrodes from sparse montages. Although computationally efficient and geometrically principled, its reliance on smoothness priors limits performance in realistic low-density settings. In particular, SSI often fails to capture subject-specific dynamics and rapidly evolving rhythms, leading to artifacts and reduced cross-subject generalization.

The advent of generative adversarial networks marked a shift toward data-driven reconstruction. EEGSR-GAN (Corley and Huang 2018) introduced an adversarial framework in which a generator synthesizes high-density EEG from sparse inputs under the supervision of a discriminator, reporting substantial reductions in mean squared error relative to linear interpolation while preserving downstream task performance, such as mental imagery classification. Subsequently, DeepCNN (Han et al. 2018) proposed end-to-end convolutional architectures tailored for spatial upsampling, directly regressing low-resolution (LR) montages (e.g. 10–20) to high-resolution (HR) layouts (e.g. 10–10 or geodesic grids). These models effectively preserve sensor-level correlations and improve source localization accuracy in paradigms such as auditory oddball tasks, yet may exhibit temporal smoothing that attenuates fast dynamics. DeepEEGSR (Tang et al. 2023) extended this paradigm by introducing structured spatio-temporal masking schemes—particularly Case 1, which emulates realistic LR acquisition by masking subsets of HR electrodes while preserving temporal continuity. This strategy enables robust supervision from high-density datasets and narrows the performance gap between laboratory-grade and portable EEG systems, as reflected in improved BCI metrics such as kappa scores.

Transformer-based approaches subsequently incorporated global attention mechanisms into EEG SR. ESTFormer (Li et al. 2025) uses spatiotemporal transformers with learnable mask tokens to capture long-range dependencies across channels and time, outperforming convolutional baselines in reconstructing oscillatory activity on benchmarks such as SEED. Hybrid EstFormer+CNN variants combine self-attention with local convolutions to balance global context modelling and computational efficiency. MASER (Zhang et al. 2024) represents a state-space alternative, integrating Mamba blocks for selective state propagation along sequences. This design improves normalized MSE and Pearson correlation on SEED and motor imagery datasets, particularly for non-stationary rhythms relevant to affective and cognitive decoding, while retaining favorable scaling properties (Zitnik et al. 2024, Lomoio et al. 2025).

More recently, diffusion models have reframed EEG SR as probabilistic generative modelling. STAD (Wang et al. 2025) (Spatio-Temporal Adaptive Diffusion) conditions reverse diffusion on LR inputs through multi-scale transformers, enabling 64-to-256 channel reconstruction with state-of-the-art gains in classification and inverse source modelling. SRGDiff (Liu et al. 2025) introduces step-aware residual guidance, adaptively weighting LR information across denoising steps to improve temporal–spatial fidelity over GAN and diffusion based baselines.

Despite these advances, key limitations remain. Diffusion-based methods typically require multi-step sampling (often 50–100 iterations), constraining real-time applicability in BCI and clinical workflows. Conversely, transformer and state-space architectures emphasize deterministic reconstruction without explicit probabilistic modelling, increasing susceptibility to mode collapse and reduced distributional diversity.

**Figure 3:**
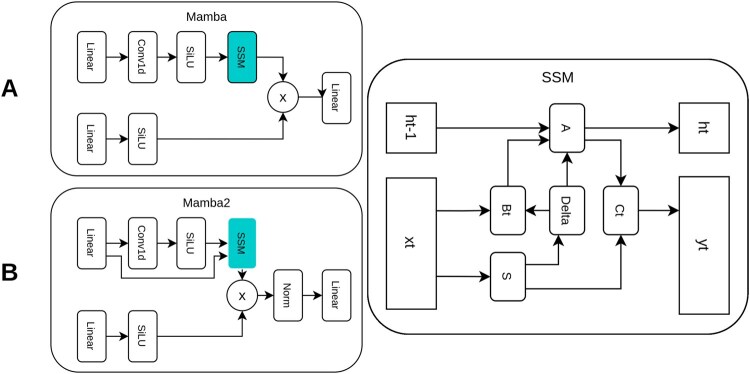
Comparison of the Functional Architectures of Mamba-1, Mamba-2, and SSM. (A) Original Mamba-1 architecture. Sequential processing through an input linear layer, Conv1D local mixing, and SiLU-gated selective state-space model (SSM) with input-dependent parameters (Δ, B, C). Dual SiLU activations frame the recurrent core, enabling efficient linear-time modelling of long sequences such as EEG signals. (B) Mamba-2 architecture. Adds layer normalization after the SSM and gating (×) for hardware-optimized state-space duality with larger state dimensions. On the right a generic selective SSM block. Input-dependent Δ, B, C parameters support scalable long-sequence processing in biomedical signals, including EEG.

DiBiMa-EEGSR addresses these gaps through a bidirectional Mamba—augmented diffusion architecture that enables one-step HR synthesis from LR inputs as depicted in Fig. 3. By training at maximal noise levels and incorporating residual guidance from upsampled signals, the framework achieves orders-of-magnitude faster inference while preserving reconstruction fidelity, thereby reconciling efficiency with spatiotemporal consistency. Moreover, DiBiMa-EEGSR extends EEG SR to temporal upsampling, elevating low-sampling-rate recordings (e.g. 40 Hz) to higher rates (e.g. 160 Hz) via model-guided interpolation within a one-step diffusion process. This problem is intrinsically more challenging than analogous tasks in quasi-periodic biosignals such as ECG (Lin et al. 2025), as EEG dynamics are inherently aperiodic and non-stationary, lacking stable harmonic structure to support straightforward extrapolation.

### 1.2 Problem formulation

Let $X^{\text{LR}}\in R^{C_{L}\times T}$ denote a low-resolution (LR) EEG recording acquired using $C_{L}$ electrodes over *T* time samples, where $C_{L}\ll C_{H}$. The corresponding high-resolution (HR) EEG signal is denoted as $X^{\text{HR}}\in R^{C_{H}\times T}$, recorded using a dense electrode configuration with $C_{H}$ channels. The objective of spatio-temporal EEG super-resolution is to learn a mapping function $f_{\theta }(\cdot )$ parameterized by θ that reconstructs $X^{\text{HR}}$ from $X^{\text{LR}}$:


(1) $${\hat{X}}^{\text{HR}}=f_{\theta }\left(X^{\text{LR}}\right),$$

where ${\hat{X}}^{\text{HR}}$ denotes the reconstructed HR EEG signal.

Due to the ill-posed nature of the super-resolution problem, multiple HR realizations may correspond to the same LR observation. To address this ambiguity, the reconstruction task is formulated within a probabilistic generative framework. Specifically, the goal is to model the conditional distribution $p(X^{\text{HR}}|X^{\text{LR}})$, which captures the underlying spatio-temporal structure and uncertainty of HR EEG signals conditioned on sparse observations. This formulation enables the generation of physiologically plausible HR reconstructions that are consistent with the observed LR measurements (Zhang et al. 2025) (Fig. 3).

In this work, we adopt a diffusion-based generative modeling approach to approximate the conditional distribution $p(X^{\text{HR}}|X^{\text{LR}},P_{\mathrm{elec}},L_{\mathrm{label}})$. A forward diffusion process progressively perturbs HR EEG signals by adding Gaussian noise over a sequence of diffusion steps $t=1,\ldots ,T_{d}$, transforming $X^{\text{HR}}$ into a noisy latent representation. The corresponding reverse process is learned to iteratively denoise the latent variables and recover HR EEG samples conditioned on the LR input. Formally, the denoising network $\epsilon_{\theta }(\cdot )$ is trained to estimate the added noise at each diffusion step:


(2) $$L_{\text{diff}}=E_{X^{\text{HR}},X^{\text{LR}},t,P_{\text{elec}},L_{\text{label}},\epsilon }\left[{\left|\epsilon -\epsilon_{\theta }(X_{t}^{\text{HR}},X^{\text{LR}},P_{\text{elec}},L_{\text{label}},t)\right|}_{2}^{2}\right]$$

where $X_{t}^{\text{HR}}$ denotes the noisy HR signal at diffusion step *t*, $P_{\mathrm{elec}}$ denotes electrode positions, $L_{\mathrm{label}}$ denotes sample label, and ϵ is sampled from a standard Gaussian distribution.

The denoising function $\epsilon_{\theta }(\cdot )$ is parameterized using a modified Bidirectional Mamba-2 state-space model, which explicitly captures long-range temporal dependencies and inter-channel spatial relationships in EEG signals. The bidirectional formulation allows the model to leverage both past and future temporal context when reconstructing each time point, aligning with the non-causal nature of electrophysiological data. By integrating the Mamba-2 backbone within the diffusion framework, the proposed formulation enables efficient and scalable modeling of spatio-temporal EEG dynamics with linear computational complexity in sequence length.

In diffusion-based super-resolution for EEG signals, the training phase establishes the model’s ability to denoise from noised high-resolution (HR) targets back to clean signals, conditioned on low-resolution (LR) inputs—this forward noising and reverse prediction loop underpins the one-step inference capability introduced earlier.

To formalize this process, we propose Algorithm 1. It samples a batch of (lr,hr,pos,label), adds random noise to *hr* at uniform timesteps t∼U(0,T) via the scheduler (e.g. producing $x_{t}=\sqrt{{\bar{\alpha }}_{t}}hr+\sqrt{1-{\bar{\alpha }}_{t}}\epsilon$), and trains the model to predict either the noise ϵ or clean *hr* via MSE loss, with optional spatio-temporal conditioning. This aligns training with inference by exposing the model to maximum-noise regimes (t→T), enabling direct mapping from pure Gaussian noise to HR during one-step sampling while preserving EEG-specific patterns like oscillatory rhythms and spatial topography.

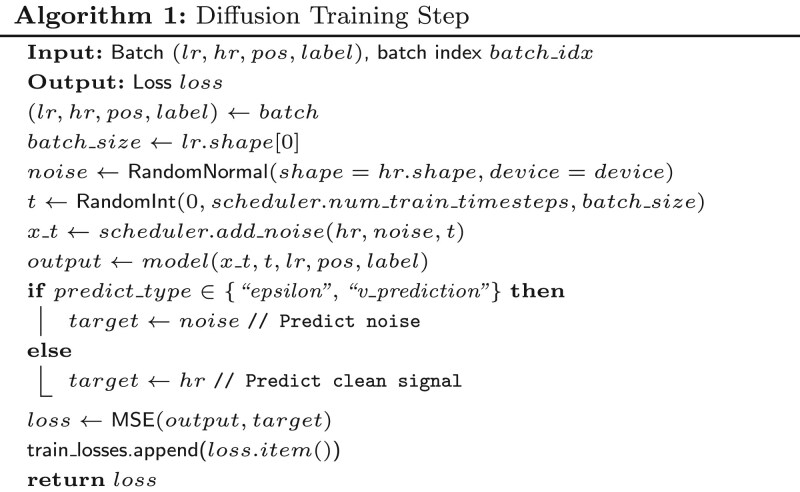

In the problem formulation, diffusion-based super-resolution (SR) for EEG signals traditionally relies on multi-step reverse processes that iteratively denoise from Gaussian noise to high-resolution (HR) outputs, conditioned on low-resolution (LR) inputs—this incurs high computational overhead unsuitable for real-time biomedical applications like brain-computer interfaces.

To address this limitation, we introduce a novel one-step sampling paradigm that directly predicts clean HR signals from noisy initials in a single model forward pass, leveraging upsampled LR as guidance while preserving spatio-temporal fidelity critical for EEG analysis. This approach aligns with the core challenge: given an LR EEG signal $lr\in R^{B\times C_{lr}\times L_{lr}}$, generate subject-specific HR output $x\in R^{B\times C_{hr}\times L_{hr}}$ (where *B* is batch size, *C* channels, *L* length) efficiently.

To formalize the one-step sampling process from upsampled LR input, we propose Algorithm 2. It first prepares optional conditioning like position encoding $\mathrm{pos}\in R^{B\times 3}$ (for spatio-temporal awareness) and class labels *label*, then upsamples *lr* via interpolation (temporal SR) or zero-padding (spatial SR) into $lr_{up}\in R^{B\times C_{hr}\times L_{hr}}$ if using residual guidance. Noise *x* is initialized as N(0,I) at the model’s target shape, optionally biased by $lr_{\mathrm{upsampled}}$, and passed through the denoiser at maximum timestep t=T−1 (e.g. 999), where the model learns to map pure noise directly to clean data conditioned on *lr*, yielding HR in one step. This bypasses iterative reverse diffusion (e.g. DDIM’s 50–100 steps), reducing inference time by orders of magnitude while incorporating LR context to ensure anatomical consistency in EEG super-resolution tasks.

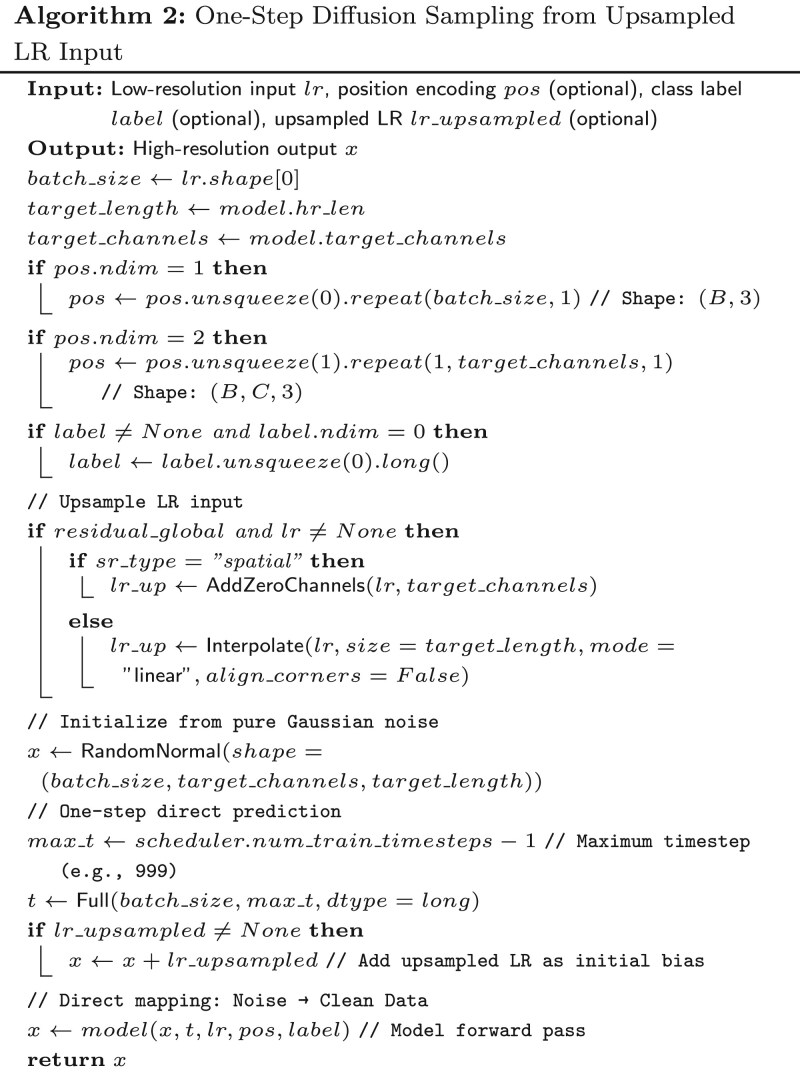

## 2 Materials and methods

### 2.1 Architecture of the proposed system

**Figure 4:**
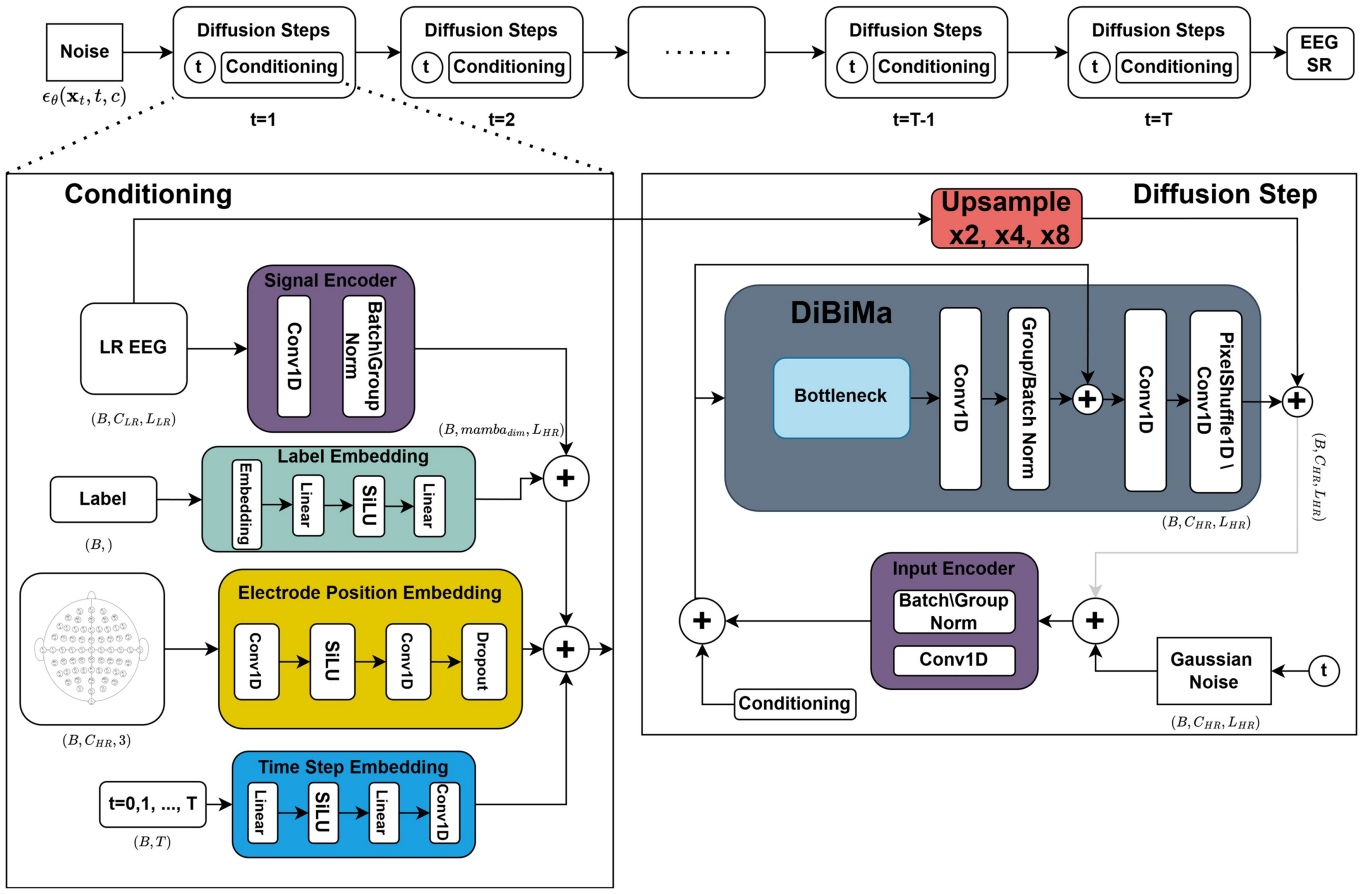
DiBiMa-EEGSR architecture and diffusion process. Forward diffusion progressively adds Gaussian noise over T=1000 timesteps via DDPM scheduler. Reverse process iteratively denoises conditioned on DiBiMa encoder features predicting high-resolution EEG.

The proposed system is a conditional Diffusion model with BiMamba-ResConv layers for temporal and spatial EEG Super-Resolution, DiBiMa-EEGSR specifically designed to handle both types of super-resolution of biomedical temporal multichannel signals like EEGs. Figure 4 depicts the model architecture.

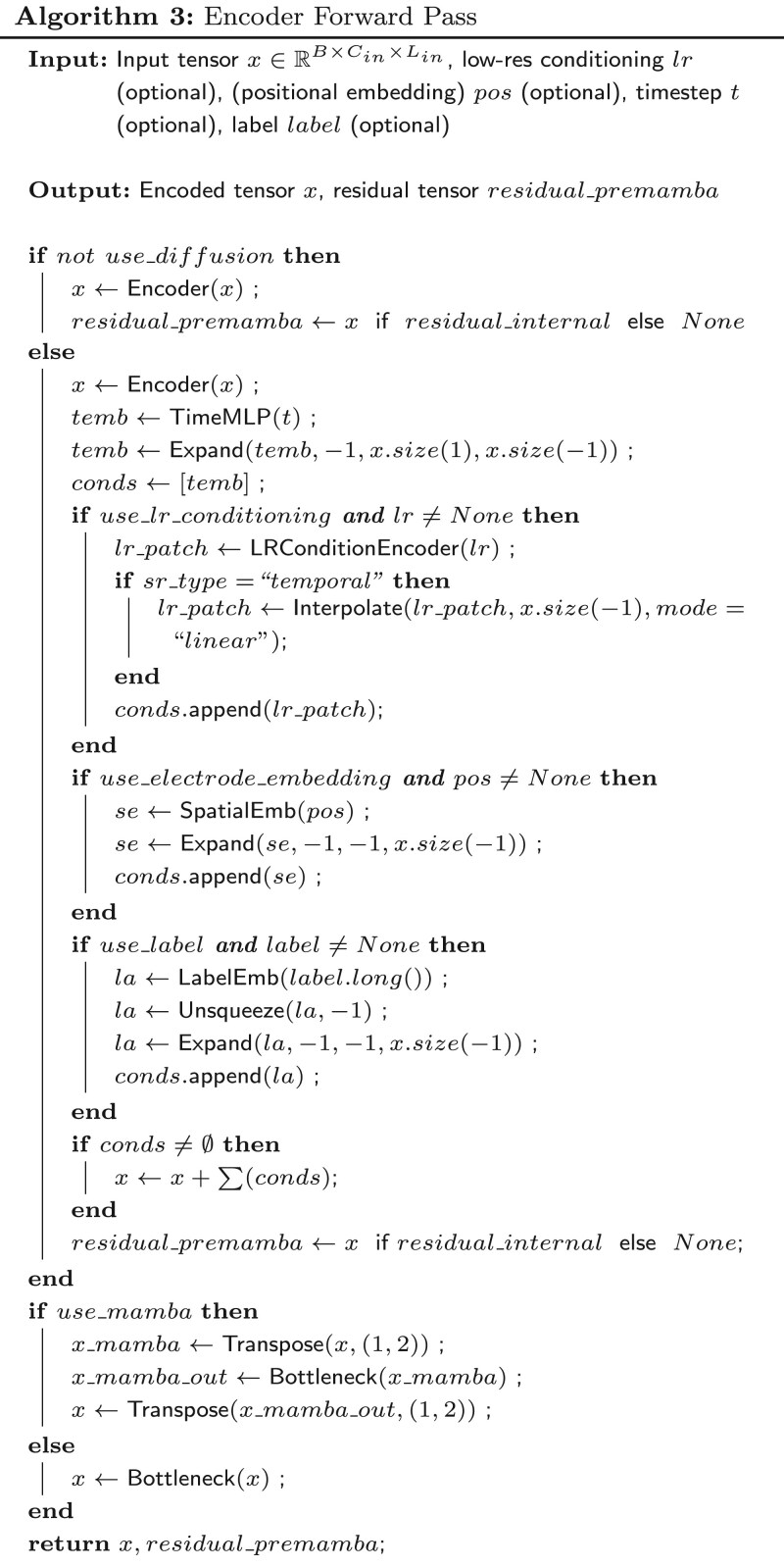

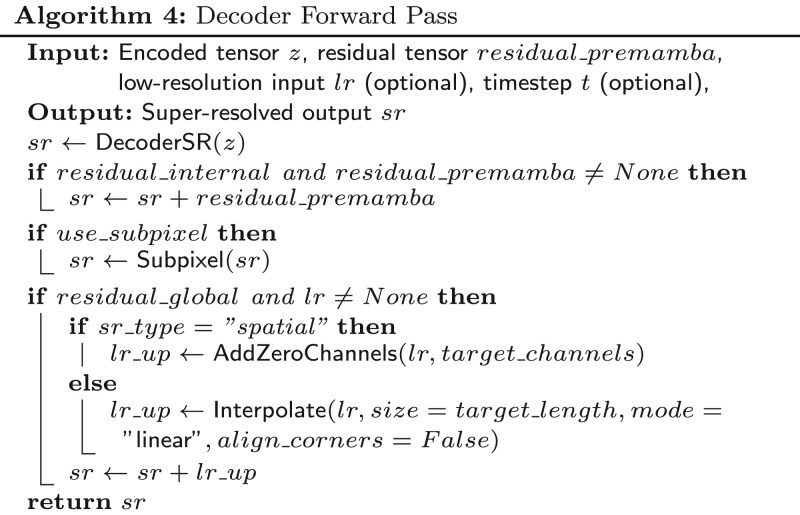

**Figure 5:**
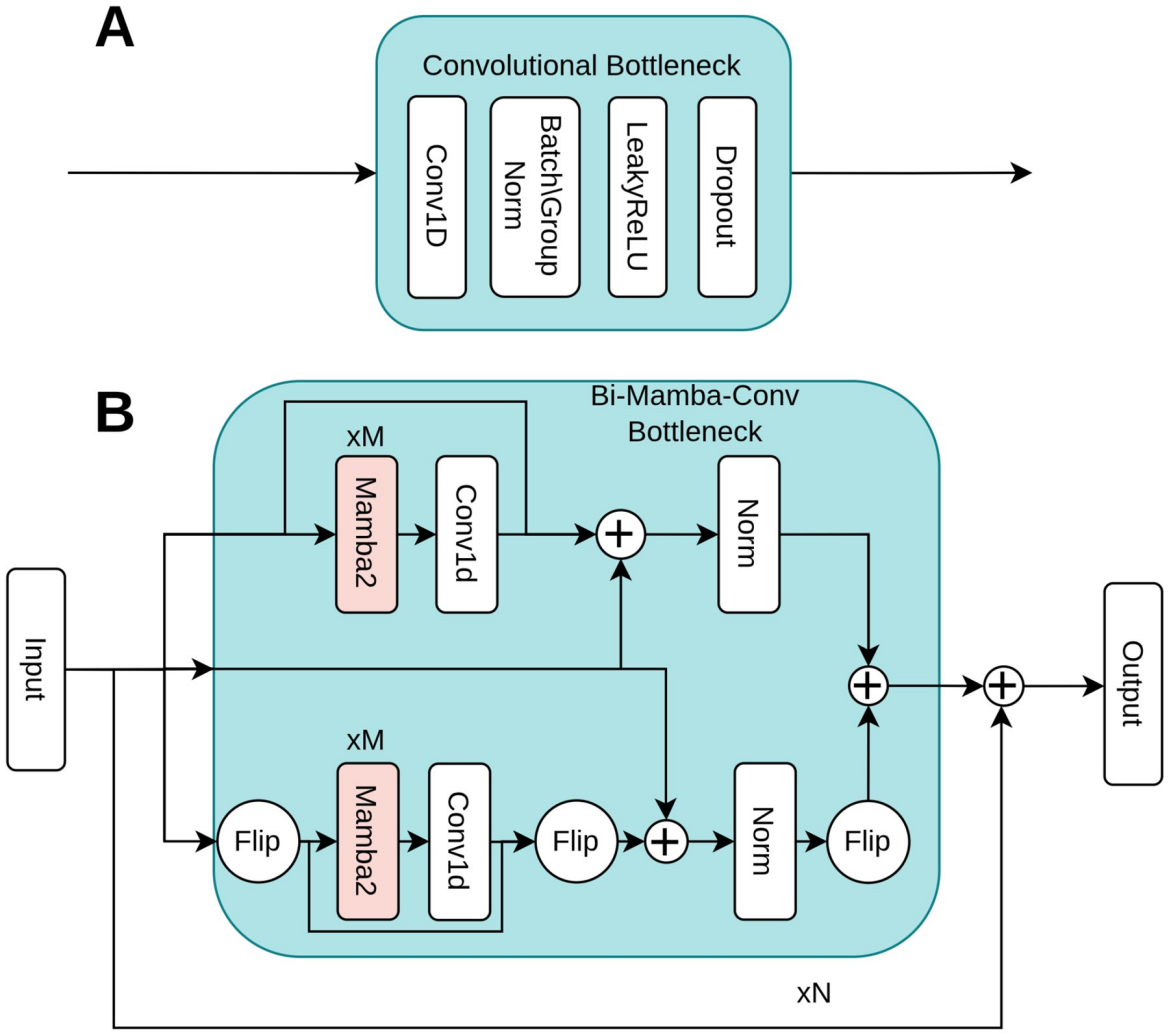
Bottlenecks of Standard Architectures and BiMamba-ResConv. (A) Standard convolutional bottleneck. Sequential Conv1D, BatchNorm, LeakyReLU, and Dropout layers enable dimensionality reduction/expansion and spatial feature extraction through fixed receptive fields. (B) BiMamba-ResConv bottleneck. Integrates bidirectional Mamba blocks for linear-time sequence modelling with skip connections and forward–backward convolutions, capturing long-range temporal dependencies and spatial super-resolution for EEG channel interpolation in DiBiMa-EEGSR.

To compare the effectiveness of Bidirectional Mamba layers bottleneck in our proposed architecture, we replaced it with a traditional Convolutional layer and compared the results obtained. Both bottlenecks are depicted in Fig. 5.

We configure the DDPM scheduler with 1000 timesteps using prediction type “sample,” a squaredcos_cap_v2 beta schedule linearly interpolated from $\beta_{0}=10^{-4}$ to $\beta_{T}=0.015$, and disabled sample clipping. Inference uses a deterministic one-step sampling strategy for rapid reconstruction.

This architecture performs temporal and spatial upsampling by factors of 2×, 4×, and 8× (e.g. increasing the sampling rate from 20 Hz to 160 Hz for 8× temporal super-resolution) and:

- Accurately reconstructs transient and localized EEG dynamics across time and space, capturing short, fast brain activity patterns without blurring important details.
- Preserves global oscillatory patterns and large-scale brain rhythms essential for understanding brain states and cognitive processes.
- Ensures spatial and temporal continuity for realistic and artifact-free EEG reconstruction, maintaining smooth and consistent signals across electrodes and time (Table 1).

**Table 1:** Dataset split for training, validation, and test sets.

Dataset	Train	Validation	Test
SEED	53 298	7614	15 236
PhysioNet M/MI	60 325	8618	17 236

To conclude the architectural overview, Table 2 summarizes the number of trainable parameters across BiMa, DiBiMa and competing models, highlighting our design’s favorable scaling properties. BiMa achieves state-of-the-art reconstruction performance with substantially fewer parameters than transformer-heavy baselines like ESTFormer and comparable with SSM models like MASER, primarily due to its bidirectional Mamba blocks and lightweight fusion modules that efficiently capture long-range temporal dependencies without quadratic complexity. This parameter efficiency—over 94.12% reduction relative to ESTFormer—translates to faster inference and lower memory footprint, making BiMa particularly suitable for deployment on edge devices and real-time EEG processing pipelines. These characteristics position BiMa as a practical advancement for resource-constrained biomedical applications while maintaining superior super-resolution fidelity. DiBiMa also outperforms other state-of-the-art methods in spatial EEG super-resolution, though it trails slightly behind our prior BiMa-EEGSR baseline. This gap likely arises because the hyperparameters optimized for BiMa’s regressive super-resolution are suboptimal for DiBiMa’s diffusion-based formulation, compounded by the fair-comparison training setup (30 epochs for BiMa versus 50 for DiBiMa). Further hyperparameter tuning or extended diffusion-specific scheduling could enable DiBiMa to surpass BiMa while leveraging its advanced multi-layer bidirectional architecture. However, despite this favorable parameter scaling, BiMa and DiBiMa exhibits moderately higher FLOPs (1.15G) compared to MASER (0.23G) under equivalent conditions, primarily attributable to its bidirectional scanning mechanism and multi-scale fusion modules that, while enhancing reconstruction quality at ×8 SR scales, introduce additional computational overhead during the forward pass. Compared to our models, original MASER models were trained for 800 entire epochs, for this reason training time is higher then our methods, that arrive to convergence after only 30 and 50 (for diffusion) epochs of training.

**Table 2:** Model informations: number of parameters (Millions), training time (in hours), and Flops per sample (Giga).

Name	Parameters (M)	Time (h)	Flops (G)
ESTFormer	33.7	0.5	1.5
EEGSR-GAN	16.5	9.0	3332.2
Deep-CNN	2.2	2.3	939.5
MASER	1.73	6.12	0.23
BiMa-EEGSR	1.97	0.135	1.15
DiBiMa-EEGSR	2.00	0.25	1.17

The temporal processing backbone utilizes Mamba-2 with $d_{\text{mamba}}=128$, $d_{\text{state}}=8$, comprising 2 layers each with 2 mamba blocks. The spatial super-resolution backbone uses Mamba-1 with identical $d_{\text{mamba}}=128$ but expanded $d_{\text{state}}=16$, structured as 2 layers with 3 blocks per layer. Both utilize element-wise addition fusion and internal residuals. Electrode position embeddings with learnable rank-based conditioning ensure precise spatial reconstruction while preserving *D*-dimensional channel outputs throughout the hierarchy.

### 2.2 Mamba blocks and the bidirectional mamba layer

The main temporal modelling component in DiBiMa-EEGSR is a modified Bidirectional Mamba layer. It stacks *N* bidirectional layers, each comprising *M* Mamba (or Mamba2) blocks per direction, and merges forward and backward streams additively. Given EEG sequences ($x\in R^{B\times C\times L}$ with B batches, C channels and length L), each layer processes the full window in both directions, providing bidirectional context that is critical for super-resolution of physiological time series.

To this end, we introduce BiMamba-ResConv, which augments bidirectional Mamba with residual pathways and lightweight convolutional refinement. Within each bidirectional layer, the forward stream applies *M* stacked Mamba blocks followed by a 1D convolution (kernel size k=3). The backward stream mirrors this computation by reversing the sequence along the temporal axis, applying the same block structure, and reversing back to the original order. The two directional features are then fused by element-wise addition:


(3) $$\text{output}={\text{forward}}_{f}+{\text{back}}_{f},$$

preserving dimensionality ($D_{\text{in}}=D_{\text{out}}=D$) while enabling symmetric information flow from both temporal directions. The directional Conv1d layers provide inexpensive local smoothing and feature refinement prior to fusion.

Bidirectional layers are stacked with skip connections,


(4) $$x^{\left(i+1\right)}=\text{BiMamba}\left(x^{\left(i\right)}\right)+\text{proj}\left(x^{\left(i\right)}\right),$$

where proj is implemented by a residual convolution (Conv1d, k=3) to align and enrich features passed across layers. Each Mamba block follows the canonical state-space formulation with fixed $d_{\text{conv}}=3$ and expand=2, while dataset-dependent hyperparameters (e.g. $d_{\text{model}}$, $d_{\text{state}}$, $n_{\text{layers}}$, $n_{\text{blocks}}$) are tuned and evaluated via ablations (Section 3.1). Overall, the design retains linear O(L) scaling with respect to sequence length while providing a full bidirectional receptive field, supporting physiologically coherent reconstruction of high-density EEG from sparse observations.

### 2.3 Datasets and preprocessing

We evaluate DiBiMa-EEGSR on two public EEG benchmarks: the SEED dataset (62 channels, 200 Hz) for emotion recognition and the M/MI dataset (64 channels, 160 Hz) for motor imagery. Together, these datasets span heterogeneous spatio-temporal dynamics and enable realistic sparse-to-dense super-resolution by simulating low-density montages.

All recordings are processed with a standardized MNE-Python pipeline tailored to dataset-specific artifacts. We assign the standard 10–10 montage with an explicit head coordinate frame. For SEED, trials with >30% corrupted channels are automatically rejected, where bad channels are defined as either flatlines (channel variance $<10^{-10}$) or saturations (consecutive differences $<10^{-8}$). Remaining bad channels are interpolated via spherical splines. Both datasets are then denoised using a 50 Hz notch filter followed by FIR bandpass filtering with dataset-specific passbands (M/MI: 0.5–40 Hz; SEED: 1–50 Hz). SEED signals are further normalized using per-channel z-scoring,


(5) $${\hat{x}}_{c}=\frac{x_{c}-\mu_{c}}{\sigma_{c}+10^{-8}},$$

with an optional common average reference.

Trials are segmented into 2 s windows and split into 80/10/10 train/validation/test partitions. For spatial super-resolution, we adopt the spatio-temporal channel masking protocol and explicitly use the Case 1 configuration of (Tang et al. 2023) to emulate practical low-density acquisition while preserving temporal continuity.

### 2.4 Metrics

BiMa and DiBiMa EEGSR reconstruction quality is comprehensively evaluated using signal fidelity, structural preservation, and noise resilience metrics standard in EEG super-resolution literature. These quantify both point-wise accuracy and physiological plausibility across sparse-to-dense channel interpolation.

Pixel-level fidelity is measured via Normalized Mean Squared Error (NMSE), which normalizes reconstruction error by signal energy to enable dataset comparison:


$$\text{NMSE}(\hat{X},X)=\frac{|\hat{X}-X{|}_{2}^{2}}{|X{|}_{2}^{2}}$$

The complementary Pearson Correlation Coefficient (PCC) assesses linear similarity per channel, then averaged across the montage to capture spatial consistency:


$$\text{PCC}({\hat{X}}_{c},X_{c})=\frac{\text{cov}(\hat{X}c,X_{c})}{\sigma \hat{X}c\sigma X_{c}}$$

Perceptual and structural quality leverages Structural Similarity Index (SSIM), emphasizing luminance, contrast, and phase alignment critical for topographic EEG maps:


$$\text{SSIM}(\hat{X},X)=\frac{\left(2\mu_{\hat{X}}\mu_{X}+c_{1})(2\sigma_{\hat{X}X}+c_{2}\right)}{\left(\mu_{\hat{X}}^{2}+\mu_{X}^{2}+c_{1})(\sigma_{\hat{X}}^{2}+\sigma_{X}^{2}+c_{2}\right)}$$

Noise robustness is evaluated through Peak Signal-to-Noise Ratio (PSNR) with MAX=1 for normalized signals and Signal-to-Noise Ratio (SNR):


$$\text{PSNR}=10{\;\text{log}\;}_{10}\left(\frac{1}{\text{MSE}}\right),\text{SNR}=10{\;\text{log}\;}_{10}\left(\frac{|X{|}_{2}^{2}}{|\hat{X}-X{|}_{2}^{2}}\right)$$

Electrode topology is assessed via topographic smoothness (discrete Laplacian on reconstructed montage), ensuring anatomically coherent interpolation. Physiological fidelity validates PSD bandpower preservation via per-band Mean Absolute Error (MAE) and imaginary coherence between reconstructed channel pairs.


$$MAE_{i}=|\;\text{log}({\hat{P}}_{i})-\text{log}(P_{i})|\;\text{for}\;i\in \{\delta ,\theta ,\alpha ,\beta ,\gamma \}$$

Downstream utility is measured by classification accuracy on motor imagery (M/MI) using ResNet50 classifier to predict open-close eye using both low-high-super resolution signals and explain the predictions using GradCam++.

## 3 Results

This section presents the evaluation of DiBiMa-EEGSR across two benchmark datasets (SEED and MM/MI) for both spatial (i.e. 8→64 channels) and temporal (i.e. 200→1600 samples) EEG super-resolution tasks. We first conduct systematic ablation studies to isolate and quantify the contribution of individual architectural components: the presence of Bidirectional Mamba blocks versus diffusion modeling, Mamba hyperparameters (d_model, d_state, n_blocks, n_layers), diffusion conditioning strategies (from no conditioning to full LR + electrode + label conditioning), and sampling initialization approaches (pure noise versus upsampled LR + noise). These ablations establish the necessity of each design choice and identify optimal configurations. Following the ablation analysis, we report final results comparing DiBiMa-EEGSR and BiMa-EEGSR (same Bidirectional Mamba model with no diffusion) against state-of-the-art baselines across all metrics (MSE, RMSE, PSNR, SSIM, NMSE, PCC, SNR, NRMSE), demonstrating both quantitative improvements and qualitative gains in physiological signal fidelity.

### 3.1 Ablation studies

Ablation studies were performed by examining : (i) the presence of Mamba blocks, (ii) the variation of key architectural hyperparameters, (iii) the conditioning strategies of the diffusion model, and (iv) sampling initialization approaches. These experiments isolate the impact of temporal modeling capacity and generative modeling design on spatial and temporal EEG super-resolution quality.

Four model configurations across both SEED and MM/MI datasets for spatial (8→64 channels) and temporal (200→1600 samples) super-resolution tasks have been used:

- Full model (Mamba ✓, Diffusion ✓): Complete DiBiMa-EEGSR architecture;
- Diffusion-only (Mamba ✗, Diffusion ✓): Encoder-decoder with diffusion process but without bidirectional temporal modeling;
- Mamba-only (Mamba ✓, Diffusion ✗): Deterministic regression with bidirectional Mamba layers, also referenced as BiMa-EEGSR;
- Baseline (Mamba ✗, Diffusion ✗): Pure encoder-decoder without advanced components.

Each configuration was trained for 30 epochs using the same preprocessing, optimizer settings, and evaluation protocols to ensure fair comparison. Performances were quantified using MSE, RMSE, PSNR, SSIM, NMSE, PCC, SNR metrics.

We systematically varied four key Mamba hyperparameters while holding others constant:

- mamba_version: **1**, 2—which version of Mamba to use.
- d_model (dimension): 32, **64**, 128—controls the hidden state dimensionality of each Mamba block;
- d_state (state dimension): 8, **16**—determines the SSM state space size
- n_mamba_blocks (number of blocks per layer): 1, 2, **3**—affects temporal modeling depth within each bidirectional layer

The baseline configuration (bolded) was selected based on preliminary experiments balancing reconstruction quality and computational efficiency. All experiments used the same number of bidirectional layers (n_mamba_layers: 1, **2**) and merge strategy (element-wise addition) to isolate individual parameter effects.

Ablation trials were executed using a sequential optimization strategy implemented for evaluating one hyperparameter at a time while fixing others at baseline values. Each trial used 30 training epochs on preprocessed EEG segments with consistent train/validation splits. Models were evaluated on held-out validation sets, with metrics averaged across all test samples to ensure statistical reliability.

Results are reported for both datasets (SEED: 62 channels, 200 Hz; MM/MI: 64 channels, 160 Hz) to assess generalization across different EEG recording protocols and cognitive tasks.

To evaluate diffusion model design choices, we conducted ablation studies on both the conditioning inputs to the denoiser and the sampling initialization strategy. These experiments quantify how progressively richer conditioning signals affect EEG super-resolution quality in both spatial and temporal settings.

To evaluate diffusion model design choices, we conducted ablation studies on both the conditioning inputs to the denoiser and the sampling initialization strategy. These experiments quantify how progressively richer conditioning signals affect EEG super-resolution quality in both spatial and temporal settings.

**Figure 6:**
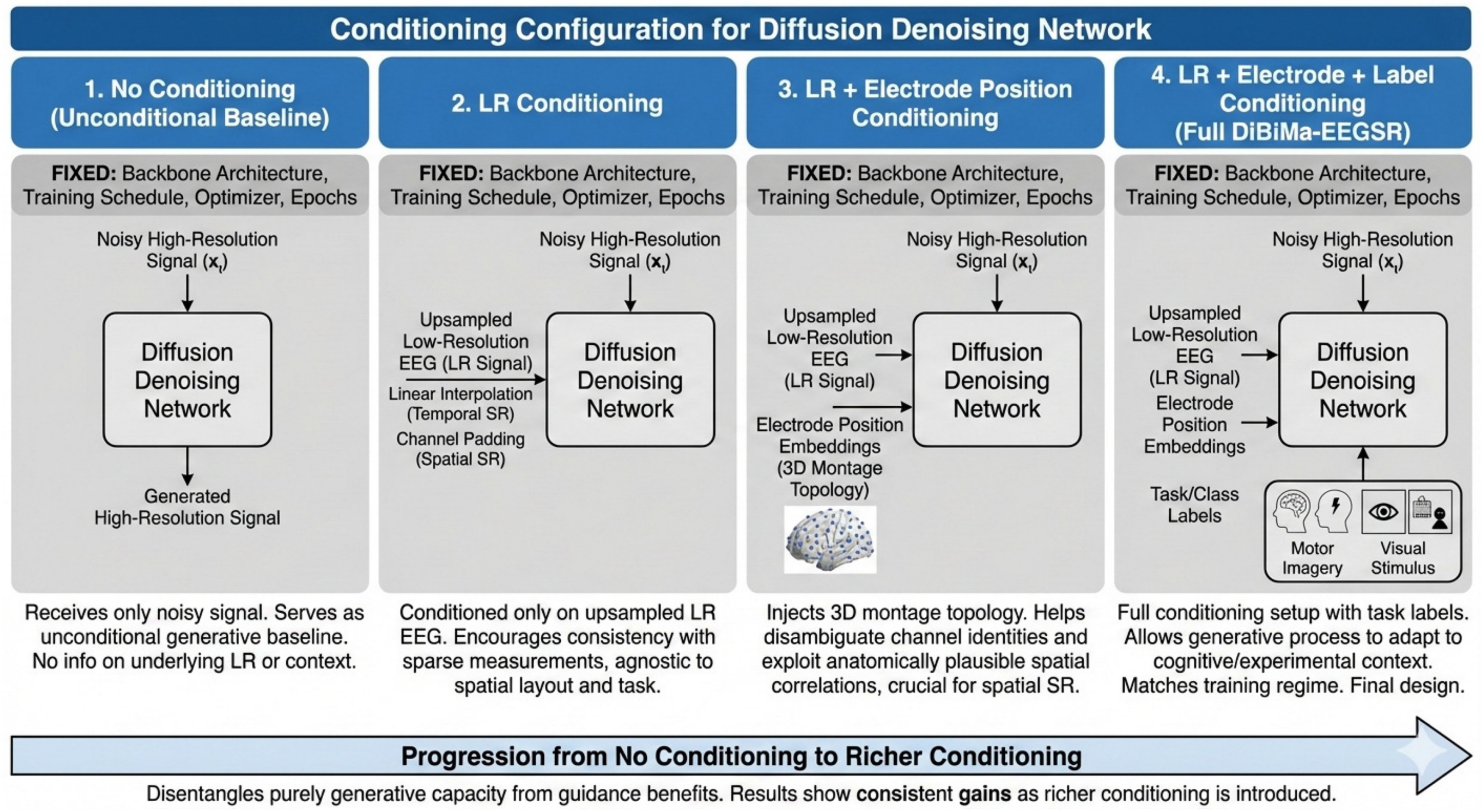
Different conditioning configurations proposed for DiBiMa-EEGSR. Four architectures progress from left to right: (1) No conditioning with fixed denoising schedules and optimizers on noisy HR EEG signals, generating HR signals without LR context; (2) 2D LR signal conditioning improves spectrum alignment and agnostic-to-LR robustness; (3) LR electrode position conditioning extracts anatomically plausible spatial topology; (4) LR electrode + label conditioning enables full cognitive state-aware training. Progressive gains demonstrate richer conditioning benefits.

Using the approach depicted in Fig. 6, we compared four conditioning configurations for the diffusion denoising network, keeping the backbone architecture and training schedule fixed:

- No conditioning: The denoiser receives only the noisy high-resolution signal $x_{t}$, without any information about the underlying low-resolution observation or experimental context. This setting serves as an unconditional generative baseline.
- LR conditioning: The denoiser is conditioned only on the upsampled low-resolution EEG, concatenated or added as an extra input stream depending on the task (linear interpolation for temporal SR, channel padding for spatial SR). This encourages the model to reconstruct signals consistent with the observed sparse measurements while remaining agnostic to spatial layout and task labels.
- LR + electrode position conditioning: In addition to the upsampled LR signal, we inject electrode position embeddings that encode the 3D montage topology. This conditioning helps the model disambiguate channel identities and exploit anatomically plausible spatial correlations, which is particularly important for 8→64 channel spatial super-resolution.
- LR + electrode position + label conditioning: The full conditioning setup adds task or class labels on top of LR and electrode embeddings, allowing the generative process to adapt to cognitive or experimental context. This configuration matches the training regime, where the model learns to denoise $x_{t}=x_{HR}+\varepsilon$ given the triplet (LR signal, electrode positions, label), and serves as the final DiBiMa-EEGSR design.

Across all four setups, we use the same diffusion schedule, optimizer, and training epochs. The progression from no conditioning to LR + electrode + label conditioning allows us to disentangle the contribution of purely generative capacity from the benefits of subject- and task-specific guidance, and the results (reported in the ablation tables) show consistent gains as richer conditioning is introduced.

During inference, we further ablated the sampling initialization strategy, comparing:

- Pure noise initialization, where sampling starts from $x_{T}∼N(0,I)$ regardless of the LR input.
- Upsampled LR + noise initialization, where sampling starts from $x_{T}=\text{upsample}(x_{LR})+N(0,\sigma^{2}I)$, aligning the inference distribution with the training regime and leveraging the LR signal as a strong prior.

**Figure 7:**
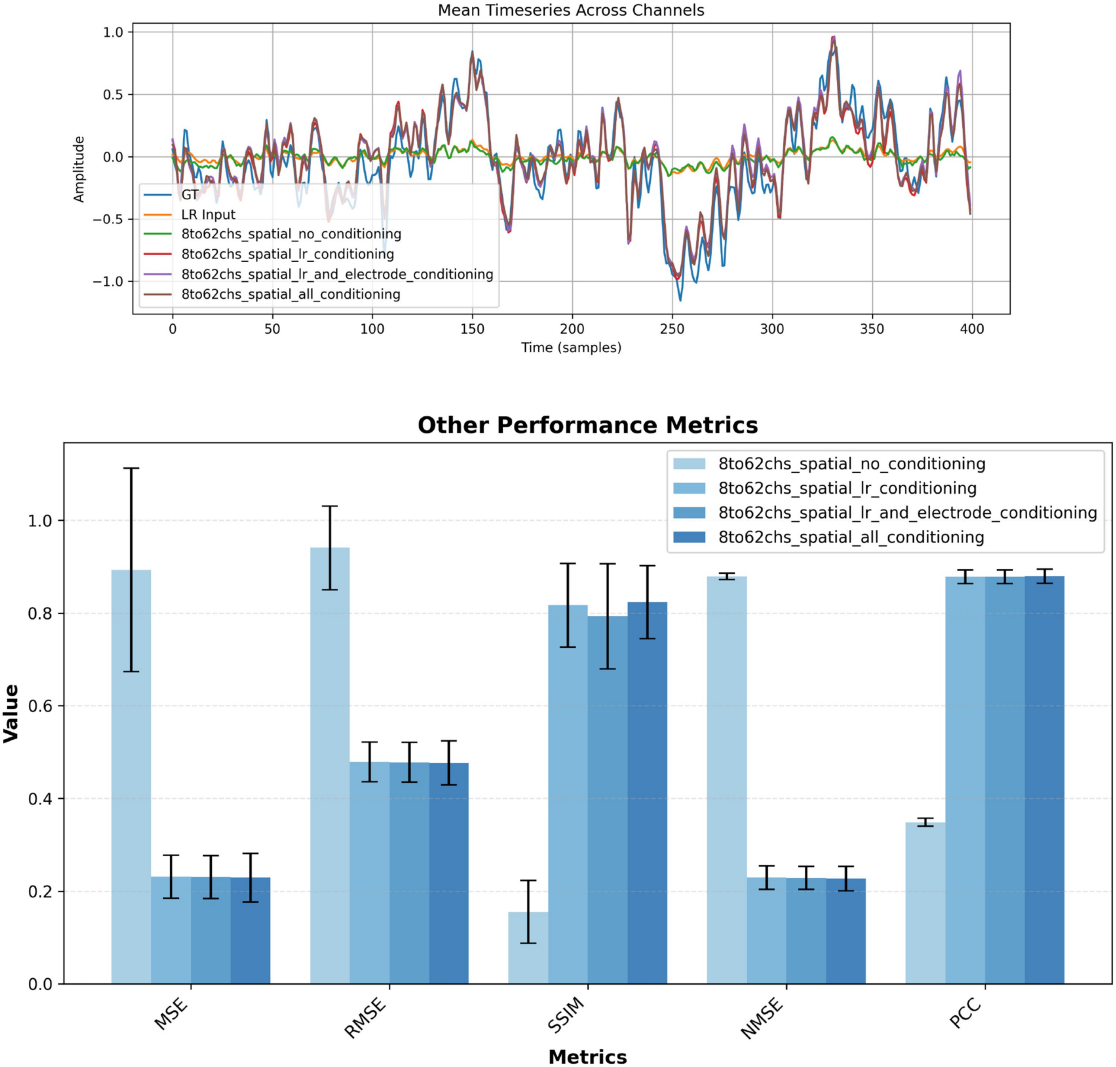
Reconstruction example for x8 spatial super-resolution on SEED dataset with corresponding evaluation metrics.

**Figure 8:**
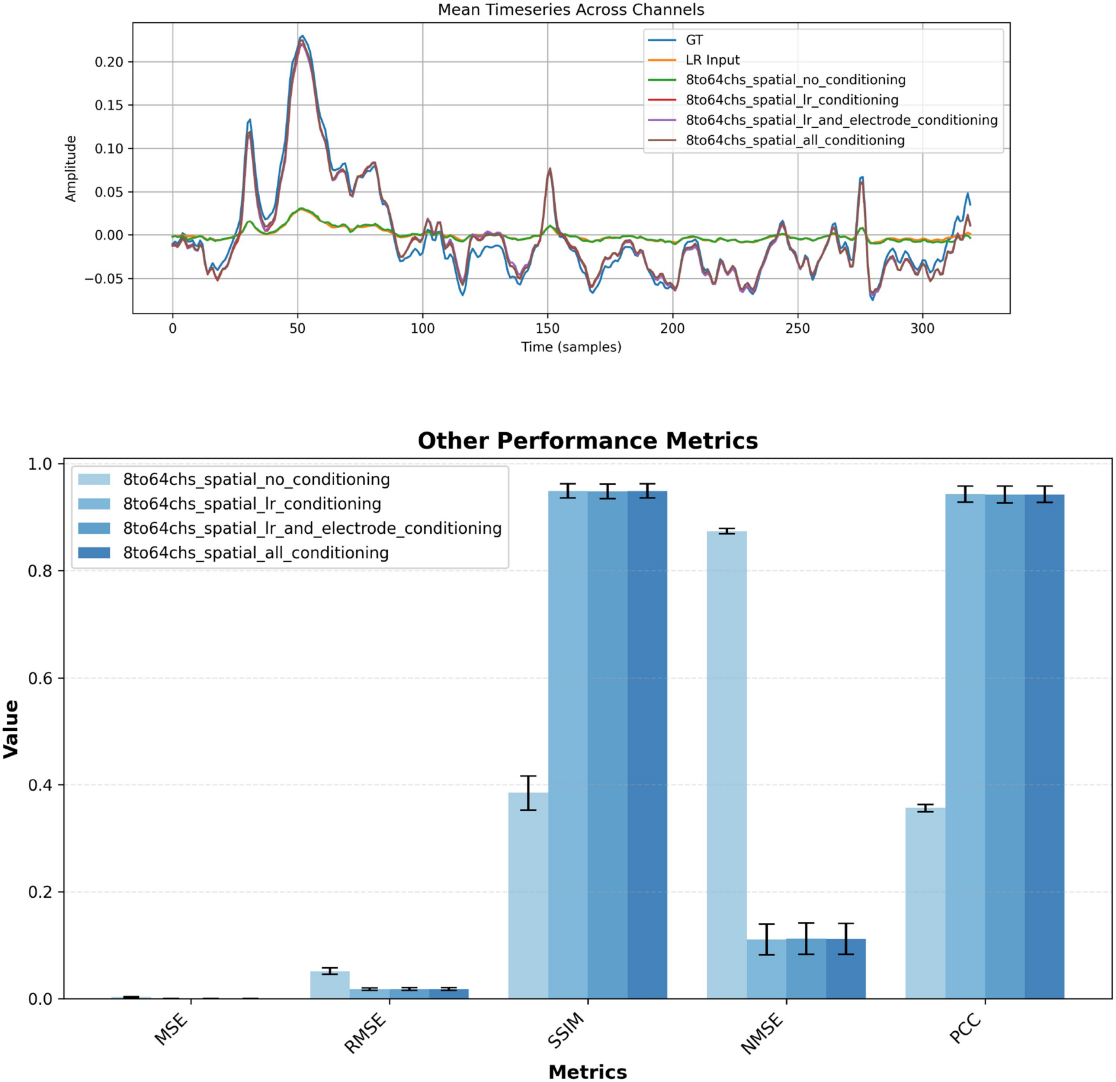
Reconstruction example for x8 spatial super-resolution on M/MI dataset with corresponding evaluation metrics.

Combined with the conditioning ablations, this grid of experiments clarifies that both informative conditioning (LR, electrode positions, labels) and LR-based initialization are crucial to obtain stable, physiologically plausible high-density EEG reconstructions (Figs 7 and 8, Tables 3–7).

**Table 3:** Ablation study on Mamba hyperparameters for EEG super-resolution (spatial & temporal) across SEED and PhysioNet M/MI datasets.^a^

Dataset	Hyperparam	Value	PSNR ↑	SSIM ↑	NMSE ↓	PCC ↑	SNR ↑
**Spatial super-resolution**							
PhysioNet M/MI	Mamba Version	**1**	34.37 ± 1.05	0.94 ± 0.01	0.1250 ± 0.031	0.9353 ± 0.017	9.16 ± 1.06
		2	34.29 ± 1.05	0.94 ± 0.01	0.1271 ± 0.032	0.9344 ± 0.017	9.09 ± 1.05
	Mamba Dimension	32	34.20 ± 1.06	0.94 ± 0.01	0.1297 ± 0.032	0.9329 ± 0.017	9.00 ± 1.06
		64	34.33 ± 1.05	0.94 ± 0.01	0.1260 ± 0.032	0.9348 ± 0.017	9.13 ± 1.06
		**128**	34.45 ± 1.05	0.94 ± 0.01	0.1226 ± 0.031	0.9366 ± 0.016	9.25 ± 1.07
	State	8	34.20 ± 1.02	0.94 ± 0.01	0.1224 ± 0.031	0.9367 ± 0.017	9.25 ± 1.07
		**16**	34.46 ± 1.06	0.94 ± 0.01	0.1227 ± 0.031	0.9366 ± 0.017	9.25 ± 1.07
	Blocks	1	34.46 ± 1.06	0.94 ± 0.01	0.1224 ± 0.031	0.9367 ± 0.017	9.26 ± 1.07
		2	34.62 ± 1.07	0.94 ± 0.01	0.1183 ± 0.031	0.9389 ± 0.017	9.42 ± 1.10
		**3**	34.66 ± 1.06	0.95 ± 0.01	0.1170 ± 0.031	0.9396 ± 0.016	9.46 ± 1.10
	BiMamba Layers	1	34.66 ± 1.05	0.95 ± 0.01	0.1170 ± 0.030	0.9396 ± 0.016	9.46 ± 1.09
		**2**	34.86 ± 1.04	0.94 ± 0.01	0.1118 ± 0.029	0.9425 ± 0.015	9.66 ± 1.09
SEED	Mamba Version	1	6.44 ± 0.68	0.81 ± 0.10	0.2328 ± 0.023	0.8760 ± 0.013	6.35 ± 0.43
		**2**	6.51 ± 0.68	0.82 ± 0.09	0.2287 ± 0.022	0.8783 ± 0.013	6.43 ± 0.42
	Mamba Dimension	32	6.42 ± 0.68	0.79 ± 0.12	0.2334 ± 0.024	0.8754 ± 0.013	6.34 ± 0.43
		64	6.51 ± 0.68	0.80 ± 0.11	0.2289 ± 0.023	0.8781 ± 0.013	6.43 ± 0.43
		**128**	6.57 ± 0.66	0.82 ± 0.08	0.2256 ± 0.022	0.8799 ± 0.012	6.48 ± 0.42
	State	8	6.47 ± 0.67	0.81 ± 0.11	0.2310 ± 0.023	0.8769 ± 0.013	6.38 ± 0.43
		**16**	6.51 ± 0.69	0.50 ± 0.27	0.2289 ± 0.023	0.8782 ± 0.013	6.43 ± 0.43
	Blocks	1	6.54 ± 0.67	0.81 ± 0.10	0.2271 ± 0.022	0.8792 ± 0.012	6.46 ± 0.42
		**2**	6.59 ± 0.66	0.82 ± 0.09	0.2246 ± 0.022	0.8806 ± 0.012	6.51 ± 0.42
		3	6.52 ± 0.67	0.74 ± 0.16	0.2284 ± 0.023	0.8784 ± 0.013	6.43 ± 0.43
	BiMamba Layers	1	6.73 ± 0.66	0.77 ± 0.13	0.2177 ± 0.021	0.8845 ± 0.012	6.64 ± 0.42
		**2**	6.93 ± 0.67	0.83 ± 0.09	0.2078 ± 0.021	0.8901 ± 0.012	6.85 ± 0.44
**Temporal super-resolution**							
PhysioNet M/MI	Mamba Version	1	31.97 ± 0.85	0.89 ± 0.02	0.2147 ± 0.043	0.8860 ± 0.024	6.77 ± 0.86
		**2**	33.69 ± 0.79	0.93 ± 0.01	0.1449 ± 0.031	0.9245 ± 0.017	8.49 ± 0.94
	Mamba Dimension	32	32.25 ± 0.85	0.90 ± 0.02	0.2015 ± 0.042	0.8933 ± 0.023	7.03 ± 0.90
		64	33.61 ± 0.79	0.93 ± 0.01	0.1477 ± 0.032	0.9231 ± 0.017	8.41 ± 0.93
		**128**	33.99 ± 0.79	0.94 ± 0.01	0.1353 ± 0.030	0.9297 ± 0.016	8.79 ± 0.95
	State	**8**	33.79 ± 0.79	0.93 ± 0.01	0.1417 ± 0.031	0.9264 ± 0.017	8.59 ± 0.94
		16	31.43 ± 0.87	0.88 ± 0.02	0.2423 ± 0.045	0.8702 ± 0.026	6.23 ± 0.80
	Blocks	1	33.48 ± 0.79	0.93 ± 0.01	0.1521 ± 0.032	0.9206 ± 0.018	8.28 ± 0.93
		**2**	34.07 ± 0.80	0.94 ± 0.01	0.1332 ± 0.030	0.9309 ± 0.016	8.86 ± 0.95
		3	33.33 ± 0.79	0.93 ± 0.01	0.1574 ± 0.033	0.9178 ± 0.018	8.13 ± 0.92
	BiMamba Layers	1	27.95 ± 0.92	0.70 ± 0.02	0.5331 ± 0.038	0.6830 ± 0.028	2.74 ± 0.30
		**2**	31.73 ± 0.88	0.88 ± 0.02	0.2267 ± 0.044	0.8792 ± 0.025	6.53 ± 0.84
SEED	Mamba Version.	**1**	6.34 ± 0.72	0.85 ± 0.03	0.2394 ± 0.031	0.8720 ± 0.018	6.24 ± 0.55
		2	6.33 ± 0.72	0.86 ± 0.02	0.2392 ± 0.031	0.8721 ± 0.018	6.25 ± 0.58
	Mamba Dim.	32	5.96 ± 0.70	0.84 ± 0.02	0.2613 ± 0.032	0.8593 ± 0.019	5.86 ± 0.52
		64	6.35 ± 0.71	0.86 ± 0.02	0.2382 ± 0.031	0.8726 ± 0.018	6.27 ± 0.55
		**128**	6.42 ± 0.73	0.86 ± 0.02	0.2346 ± 0.031	0.8747 ± 0.018	6.33 ± 0.56
	State	8	6.47 ± 0.73	0.86 ± 0.02	0.2326 ± 0.031	0.8759 ± 0.018	6.37 ± 0.57
		**16**	6.46 ± 0.72	0.87 ± 0.02	0.2321 ± 0.031	0.8761 ± 0.018	6.38 ± 0.57
	Blocks	1	5.80 ± 0.72	0.81 ± 0.03	0.2699 ± 0.031	0.8543 ± 0.019	5.72 ± 0.49
		**2**	6.46 ± 0.73	0.86 ± 0.02	0.2324 ± 0.031	0.8760 ± 0.018	6.38 ± 0.57
		3	6.45 ± 0.73	0.85 ± 0.03	0.2330 ± 0.031	0.8756 ± 0.018	6.36 ± 0.57
	BiMamba Layers	**1**	6.49 ± 0.74	0.86 ± 0.02	0.2310 ± 0.031	0.8767 ± 0.018	6.40 ± 0.57
		2	6.49 ± 0.72	0.86 ± 0.02	0.2351 ± 0.031	0.8744 ± 0.018	6.32 ± 0.56

a Lower is better for MSE, NMSE; higher is better for PSNR, SSIM, PCC, SNR. Best results bolded. Each model is trained for 30 epochs.

**Table 4:** Ablation study showing the effect of Mamba and Diffusion components across datasets and resolution types (x8 super-resolution, 30 epochs).

Type	Dataset	Mamba	Diffusion	PSNR	SSIM	NMSE	PCC	SNR
Spatial	PhysioNet M/MI	✗	✗	33.54 ± 1.11	0.93 ± 0.02	0.1511 ± 0.038	0.9215 ± 0.021	8.34 ± 1.05
		✓	✗	34.79 ± 1.04	0.95 ± 0.01	0.1135 ± 0.029	0.9415 ± 0.016	9.56 ± 1.09
		✓	✓	34.90 ± 1.04	0.95 ± 0.01	0.1128 ± 0.027	0.9418 ± 0.014	9.70 ± 1.10
	SEED	✗	✗	6.04 ± 0.73	0.77 ± 0.12	0.2553 ± 0.027	0.8631 ± 0.016	5.92 ± 0.45
		✓	✗	6.74 ± 0.66	0.51 ± 0.26	0.2170 ± 0.021	0.8849 ± 0.012	6.66 ± 0.43
		✓	✓	6.84 ± 0.67	0.83 ± 0.08	0.2085 ± 0.024	0.8898 ± 0.014	6.84 ± 0.53
Temporal	PhysioNet M/MI	✗	✗	32.47 ± 0.85	0.90 ± 0.02	0.1918 ± 0.040	0.8990 ± 0.022	7.27 ± 0.90
		✓	✗	32.64 ± 0.84	0.91 ± 0.02	0.1844 ± 0.039	0.9028 ± 0.022	7.44 ± 0.91
		✓	✓	34.79 ± 0.84	0.95 ± 0.01	0.1130 ± 0.027	0.9417 ± 0.014	9.59 ± 1.01
	SEED	✗	✗	6.18 ± 0.70	0.85 ± 0.02	0.2475 ± 0.031	0.8673 ± 0.018	6.10 ± 0.54
		✓	✗	6.38 ± 0.72	0.86 ± 0.02	0.2365 ± 0.031	0.8736 ± 0.018	6.29 ± 0.56
		✓	✓	6.50 ± 1.15	0.85 ± 0.04	0.2276 ± 0.045	0.8786 ± 0.027	6.50 ± 0.77

**Table 5:** Ablation study on diffusion conditioning for M/MI dataset.

Type	Conditioning	x8 super resolution	
		nMSE (↓)	PCC (↑)
Spatial	No	0.8743 ± 0.005	0.3552 ± 0.007
	LR Conditioning	0.1173 ± 0.030	0.9395 ± 0.016
	…+ Electrode Positions	0.1167 ± 0.030	0.9397 ± 0.016
	…+ Label	0.1170 ± 0.030	0.9396 ± 0.016
Temporal	No	0.2462 ± 0.041	0.8681 ± 0.023
	LR Conditioning	0.1134 ± 0.027	0.9415 ± 0.014
	…+ Electrode Positions	0.1138 ± 0.027	0.9413 ± 0.014
	…+ Label	0.1130 ± 0.027	0.9417 ± 0.014

**Table 6:** Ablation study on diffusion conditioning for SEED dataset.

Type	Conditioning	x8 super resolution	
		nMSE (↓)	PCC (↑)
Spatial	No	0.8757 ± 0.005	0.3528 ± 0.007
	LR Conditioning	0.2138 ± 0.024	0.8868 ± 0.014
	…+ Electrode Positions	0.2127 ± 0.025	0.8775 ± 0.014
	…+ Label	0.2084 ± 0.024	0.8898 ± 0.014
Temporal	No	0.3653 ± 0.055	0.7962 ± 0.038
	LR Conditioning	0.2293 ± 0.051	0.8774 ± 0.032
	…+ Electrode Positions	0.2297 ± 0.049	0.8773 ± 0.029
	…+ Label	0.2275 ± 0.045	0.8786 ± 0.027

**Table 7:** Ablation study on diffusion sampling for M/MI dataset.

Type	Sampling	x8 super resolution	
		nMSE (↓)	PCC (↑)
Spatial	Noise	0.115420/0.030	0.9405/0.016
	Noise + Upsampled LR	0.115393/0.030	0.9405/0.016
Temporal	Noise	0.2265/0.041	0.8795/0.023
	Noise + Upsampled LR	0.2341/0.058	0.8749/0.035

### 3.2 Results for spatio-temporal super-resolution

**Figure 9:**
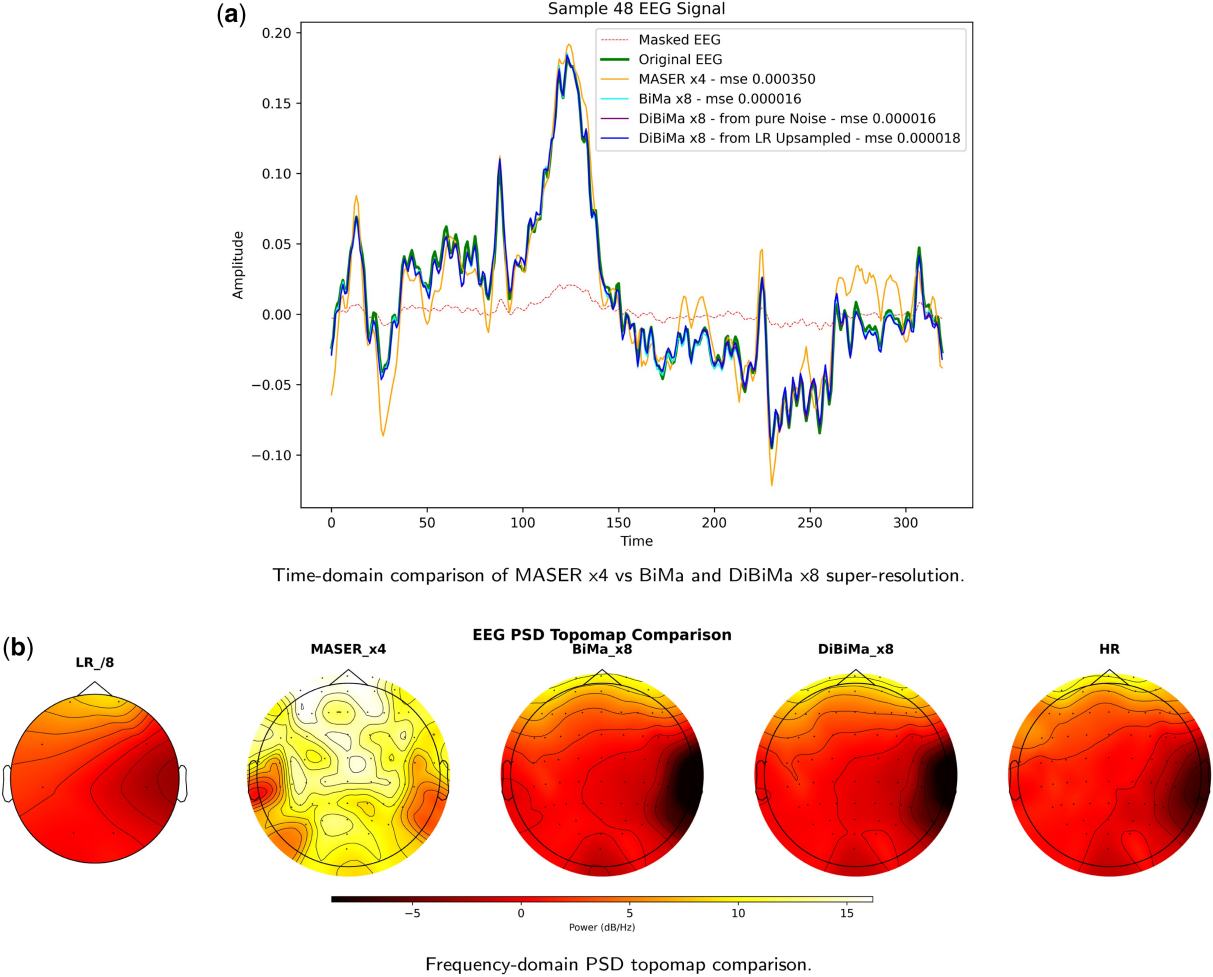
Qualitative comparison between MASER x4 against our BiMa and DiBiMa x8 super-resolution for an example signal of the M/MI dataset. Time-domain waveforms (a) show DiBiMa x8 preserving fine temporal details better than MASER x4, while PSD topomaps (b) demonstrate superior frequency-space reconstruction. (a) Time-domain comparison of MASER x4 versus BiMa and DiBiMa x8 super-resolution. (b) Frequency-domain PSD topomap comparison.

**Figure 10:**
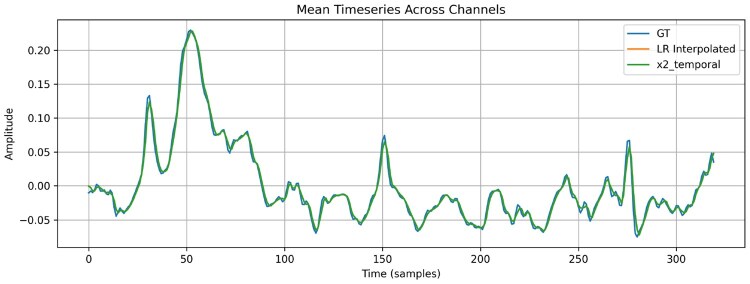
Temporal x2 super-resolution example on a random sample of the M/MI dataset.

To assess DiBiMa’s robustness and generalization, evaluations targeted an ×8 super-resolution (SR) setting, a substantially more challenging task than lower SR factors. DiBiMa (×8) outperforms MASER (×4), despite MASER’s reported ×8 results, demonstrating superior performance under stricter conditions. This underscores DiBiMa’s capacity for high-fidelity reconstruction and preservation of fine temporal-spatial details beyond state-of-the-art benchmarks. Figure 9 shows qualitative comparisons in time and frequency domains for reconstructed EEG signals from DiBiMa (×8), BiMa (×8), and MASER (×4). DiBiMa recovers fine waveform details and high-frequency components that MASER smooths out. At higher upscaling factors, DiBiMa maintains superior temporal consistency and spectral fidelity, confirming robustness across demanding SR regimes. Finally, an example of x2 temporal super-resolution on the M/MI dataset is depicted in Fig. 10.

Tables 8 and 9 provide quantitative comparisons against spatial EEG super-resolution and state-of-the-art methods for temporal, respectively, evaluating nMSE and PCC across x2, ×4 and ×8 upsampling factors for both SEED and M/MI datasets.

**Table 8:** Results under different scale factors on the PhysioNet M/MI and SEED datasets for spatial super-resolution.^a^

Dataset	Methods	Upsampling x2		Upsampling x4		Upsampling x8	
		nMSE (↓)	PCC (↑)	nMSE (↓)	PCC (↑)	nMSE (↓)	PCC (↑)
M/MI	SSI (Freeden 1984)	0.287/0.000	0.855/0.000	0.317/0.000	0.839/0.000	0.367/0.000	0.811/0.000
	EEGSR-GAN (Corley and Huang 2018)	0.224/0.000	0.887/0.001	0.258/0.002	0.860/0.001	0.308/0.003	0.832/0.003
	Deep-CNN (Han *et al.* 2018)	0.206/0.019	0.900/0.007	0.231/0.021	0.879/0.012	0.276/0.044	0.855/0.019
	Deep-EEGSR (Tang *et al.* 2023)	0.165/0.001	0.920/0.000	0.177/0.001	0.907/0.000	0.214/0.002	0.855/0.001
	ESTFormer (Li *et al.* 2025)	0.174/0.004	0.908/0.002	0.189/0.002	0.900/0.001	0.237/0.007	0.872/0.004
	ESTFormer+CNN (Li *et al.* 2025)	0.155/0.003	0.918/0.002	0.166/0.002	0.912/0.001	0.207/0.006	0.890/0.004
	MASER (Zhang *et al.* 2024)	0.109/0.004	0.960/0.006	0.131/0.005	0.928/0.004	0.155/0.006	0.896/0.005
	BiMA-EEGSR	0.0385/0.0003	0.9808/0.0001	0.0797/0.0004	0.9619/0.0003	0.1102/0.0006	0.9434/0.0003
	DiBiMA-EEGSR	0.04745/0.0004	0.9762/0.0001	0.0909/0.0036	0.9544/0.0013	0.1218/0.0040	0.9374/0.0021
SEED	SSI (Freeden 1984)	0.684/0.000	0.585/0.000	0.703/0.000	0.567/0.000	0.755/0.000	0.527/0.000
	EEGSR-GAN (Corley and Huang 2018)	0.514/0.002	0.693/0.001	0.597/0.002	0.629/0.001	0.652/0.001	0.583/0.001
	Deep-CNN (Han *et al.* 2018)	0.485/0.005	0.713/0.004	0.608/0.006	0.622/0.006	0.672/0.007	0.569/0.002
	Deep-EEGSR (Tang *et al.* 2023)	0.413/0.010	0.762/0.007	0.524/0.002	0.682/0.002	0.580/0.005	0.641/0.003
	ESTFormer (Li *et al.* 2025)	0.315/0.014	0.827/0.009	0.353/0.007	0.803/0.004	0.425/0.011	0.757/0.007
	ESTFormer+CNN (Li *et al.* 2025)	0.277/0.014	0.852/0.008	0.311/0.005	0.829/0.003	0.377/0.009	0.788/0.005
	MASER (Zhang *et al.* 2024)	0.274/0.032	0.877/0.007	0.306/0.019	0.829/0.006	0.319/0.024	0.795/0.004
	BiMA-EEGSR	0.0796/0.0003	0.9628/0.0001	0.1201/0.0003	0.9381/0.0002	0.1995/0.0005	0.8948/0.0001
	DiBiMA-EEGSR	0.1010/0.0038	0.9483/0.0020	0.1449/0.0007	0.9249/0.0003	0.2352/0.0014	0.8748/0.0007

a Train and test was repeated four times with different train-test splits.

**Table 9:** Results under different upsampling factors on the PhysioNet M/MI dataset for temporal super-resolution.^a^

Dataset	Methods	Upsampling x2		Upsampling x4		Upsampling x8	
		nMSE (↓)	PCC (↑)	nMSE (↓)	PCC (↑)	nMSE (↓)	PCC (↑)
SEED	BiMA-EEGSR	0.0181	0.9910	0.1249	0.9355	0.2205	0.8827
	DiBiMA-EEGSR	0.0194	0.9903	0.1319	0.9316	0.2349	0.8750
M/MI	BiMA-EEGSR	0.0066	0.9967	0.0523	0.9735	0.1104	0.9431
	DiBiMA-EEGSR	0.0322	0.9838	0.0532	0.9730	0.1105	0.9430

a Train and test was performed only one time.

### 3.3 Super-resolution explainability and downstream classification task

**Figure 11:**
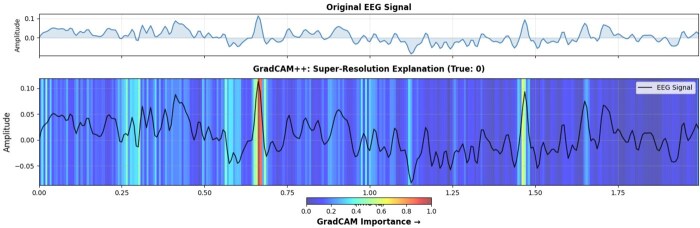
Grad-CAM++ applied to explain x8 spatial super-resolution performed by BiMa-EEGSR model.

To reveal BiMa’s decision-making in EEG super-resolution, gradient-based attribution methods—including Grad-CAM++—generate channel-wise and temporal attention heatmaps for ×2, ×4, and ×8 upsampling factors. These saliency maps show BiMa dynamically prioritizes regions with high-frequency content and cross-channel phase synchrony, essential for neurophysiological fidelity under severe downsampling (Fig. 11). This analysis validates the model’s biomedical priors and confirms its robustness in challenging regimes.

**Figure 12:**
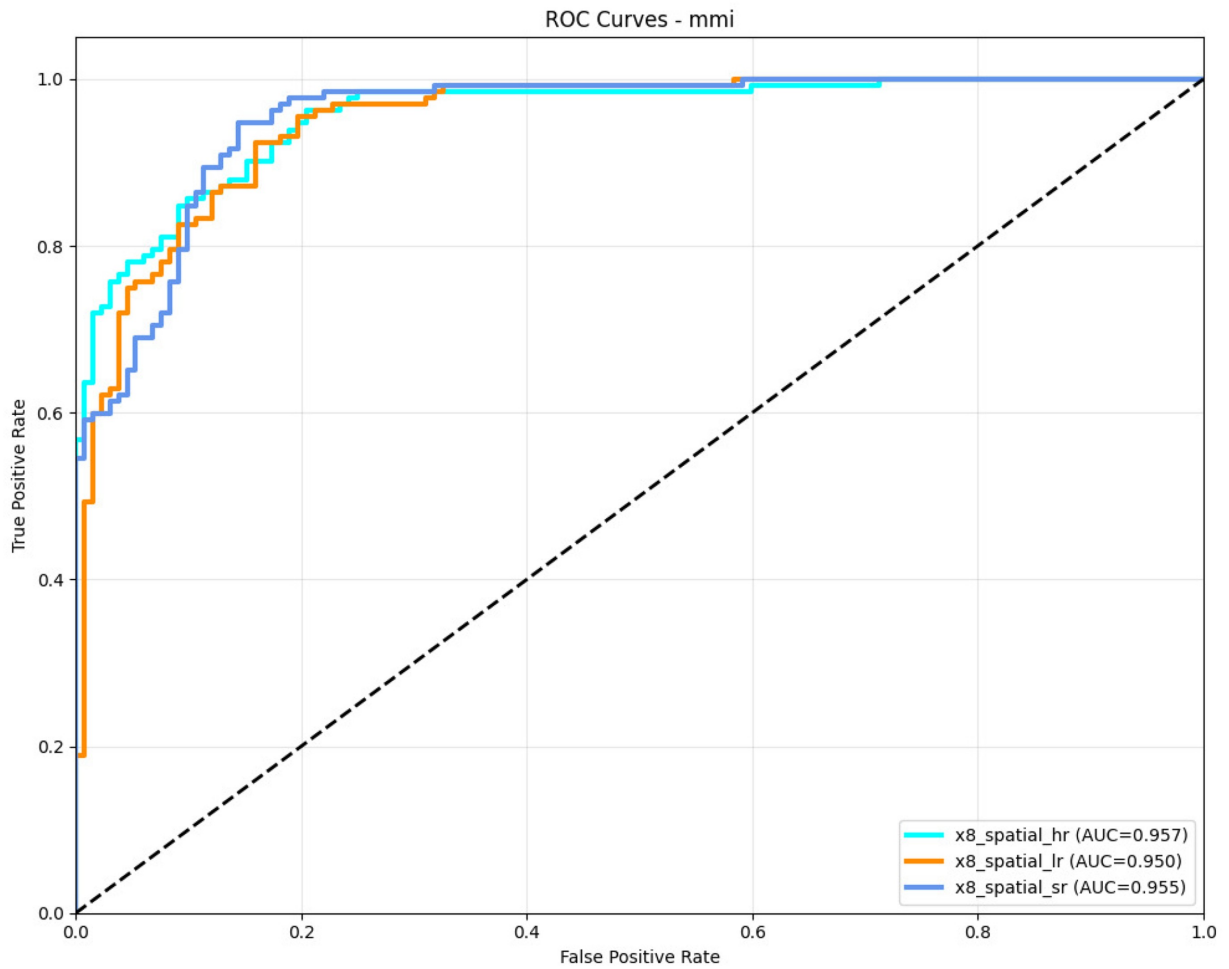
ROC-AUC curves for binary classification: LR versus SR versus HR.

The practical value of super-resolved EEG is evaluated through a binary motor imagery (MI) classification task—open versus closed eyes—using the EEGMI dataset, comparing performance across low-resolution (LR) inputs, DiBiMa super-resolved (SR) signals at ×8, and high-resolution (HR) ground truth. Quantitative results in Table 10 reveal that DiBiMa-SR substantially closes the performance gap between LR and HR baselines when using ResNet50 classifiers, with consistent gains in accuracy, F1-score, and balanced metrics. The ROC-AUC curves in Fig. 12 further illustrate that DiBiMa-SR achieves near-HR discriminative power, particularly in the high-specificity regime, underscoring effective recovery of task-relevant spatial topologies.

**Table 10:** M/MI ResNet50 classification performances on detecting open/closed eyes.

Input Type	ROC-AUC
LR	0.950
SR	0.955
HR (Ground Truth)	0.957

**Figure 13:**
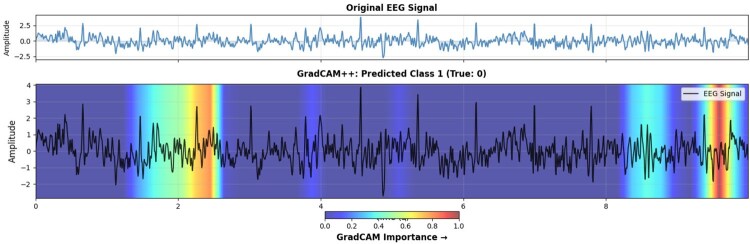
GradCAM++ heatmap highlighting key features for open-eye detection using ResNet50 classifier and BiMa-EEGSR super-resolution. Top: original mean EEG signal; bottom: GradCAM importance map showing signal regions driving the prediction.

**Figure 14:**
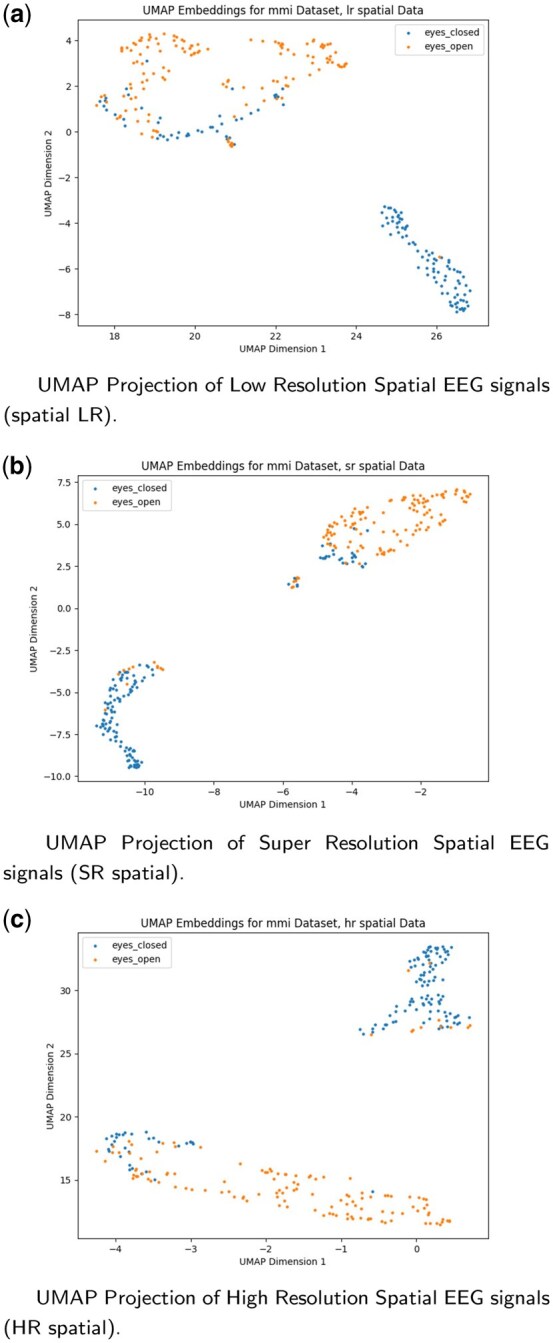
UMAP visualizations of M/MI EEG data: (A) LR, (B) SR and (C) HR spatial projections. (A) UMAP Projection of Low Resolution Spatial EEG signals (spatial LR). (B) UMAP Projection of Super Resolution Spatial EEG signals (SR spatial). (C) UMAP Projection of High Resolution Spatial EEG signals (HR spatial).

Complementing these metrics, explainability visualizations from the classifier applied to SR versus LR inputs (Fig. 13) confirm that DiBiMa enhances model focus on contralateral occipital channels (e.g. O1/O2) associated with visual imagery, mitigating the dilution of spatial discriminants in LR data. Overall, these experiments affirm DiBiMa’s dual benefits: superior reconstruction fidelity and tangible improvements in downstream neurodecoding, positioning it as a reliable preprocessing tool for resource-constrained EEG applications. We also used UMAP to visualize class-coloured embeddings on a 2D space as depicted in Fig. 14.

To evaluate generative quality across models, we computed EEG-FID scores using features extracted from a ResNet50 backbone pretrained on high-resolution EEG signals. This metric quantifies Fréchet distribution divergence in the 2048D feature space, providing a robust assessment of signal fidelity beyond pixel-level metrics.

MASER exhibited an EEG-FID of 0.2557 on the x4 spatial upsampling, reflecting its strong baseline reconstruction capabilities through state-space models. Our proposed models achieved superior performance with EEG-FID scores of 0.0064 (BiMa) and 0.0070 (DiBiMa) on the most difficult x8 spatial imputation, demonstrating enhanced preservation of spectro-temporal structure and cross-frequency interactions critical for downstream neuroscientific analysis. These results confirm our approach’s ability to generate perceptually and statistically realistic high-resolution EEG, validated against established autoencoder baselines. Table 11 reports EEG-FID metrics.

**Table 11:** EEG-FID metrics for MASER, BiMa and DiBiMa models.

Model Name	EEG-FID
MASER	0.2557
BiMa-EEGSR	**0.0064**
DiBiMA-EEGSR	0.0070

## 4 Conclusion

We presented DiBiMa-EEGSR, a unified generative framework for spatio-temporal EEG super-resolution that reconceptualizes sparse-to-dense reconstruction as structured probabilistic inference. By coupling a bidirectional state-space backbone with a conditional diffusion process grounded in low-resolution measurements, electrode geometry and task context, the model reconstructs high-density EEG while preserving waveform morphology, oscillatory dynamics and spatial structure, and remains computationally tractable for long recordings and large upsampling factors.

Across two diverse benchmarks (SEED and PhysioNet M/MI), DiBiMa-EEGSR delivered consistent gains in signal fidelity, spatial coherence and robustness relative to convolutional, transformer and existing diffusion-based baselines for both spatial (8→64 channels) and temporal (200→1600 samples) super-resolution. Systematic ablations indicate that bidirectional state-space modelling, multimodal conditioning and low-resolution–informed sampling are each critical to performance, highlighting the complementary roles of structured dynamical representations and generative denoising.

Beyond empirical improvements, our method frames EEG super-resolution as a generative inference problem, enabling uncertainty-aware reconstruction and principled incorporation of anatomical and experimental context rather than relying solely on point-estimate interpolation. In practical terms, DiBiMa-EEGSR offers a hardware-agnostic route to approximate high-density EEG from standard clinical montages, broadening access to richly resolved spatio-temporal electrophysiology in large-scale neuroscience and brain–computer interface settings where dense acquisition is impractical.

Future directions include multimodal integration with MRI and MEG, adaptive transfer across subjects and acquisition sites, and real-time or closed-loop deployment. An important open question is how generative super-resolution affects downstream analyses, including decoding performance, connectivity estimation and clinical stratification.

## Data Availability

Physionet M/MI is an open-source dataset freely available at: https://physionet.org/content/eegmmidb/1.0.0/. SEED dataset is available upon request at: https://bcmi.sjtu.edu.cn/home/seed/downloads.html

## Code availability

Code to reproduce ablation experiments, training and evaluation of the proposed BiMa and DiBiMa EEGSR models are available at https://github.com/UgoLomoio/DiBiMa-EEGSR.git. Model weights are available at https://huggingface.co/Ugo96/DiBiMa-EEGSR while an interactive demo for EEG spatial super-resolution using our models can be found at https://huggingface.co/spaces/Ugo96/DiBiMa-EEGSR-Demo.
